# Comparison of the 3D-microstructure of human alveolar and fibula bone in microvascular autologous bone transplantation: a synchrotron radiation μ-CT study

**DOI:** 10.3389/fbioe.2023.1169385

**Published:** 2023-08-25

**Authors:** Jonas Wüster, Bernhard Hesse, Rene Rothweiler, Emely Bortel, Christian Gross, Shima Bakhtiyari, Andrew King, Elodie Boller, Javier Gerber, Carsten Rendenbach, Tobias Fretwurst, Saskia Preissner, Max Heiland, Katja Nelson, Susanne Nahles

**Affiliations:** ^1^ Department of Oral and Maxillofacial Surgery, Berlin Institute of Health, Charité–Universitätsmedizin Berlin, Corporate Member of Freie Universität Berlin, Humboldt-Universität zu Berlin, Berlin, Germany; ^2^ Xploraytion GmbH, Berlin, Germany; ^3^ European Synchrotron Radiation Facility, Grenoble, France; ^4^ Department of Oral- and Craniomaxillofacial Surgery, Faculty of Medicine, Medical Center, University of Freiburg, Freiburg, Germany; ^5^ Synchrotron Soleil, Saint-Aubin, France

**Keywords:** osteocyte lacunae, bone microarchitecture, SR micro-CT, synchrotron, free fibula flap, bone vessel orientation, scaffold

## Abstract

**Introduction:** Autologous bone transplantation is successfully used in reconstructive surgery of large/critical-sized bone defects, whereby the microvascular free fibula flap is still regarded as the gold standard for the reconstruction of such defects in the head and neck region. Here, we report the morphological and lacunar properties of patient-paired bone samples from eight patients from the jaw (AB; recipient site) and the fibula (FB; donor site) on the micron length-scale using Synchrotron µ-CT. Insights into differences and similarities between these bone structures could offer a better understanding of the underlying mechanism for successful surgical outcomes and might clear the path for optimized, nature-inspired bone scaffold designs.

**Methods:** Spatial vessel-pore arrangements, bone morphology, fluid-simulation derived permeability tensor, osteocyte lacunar density, and lacunar morphology are compared.

**Results:** The orientation of the vessel system indicates a homogenous vessel orientation for AB and FB. The average mineral distance (50%) to the closest vessel boundary is higher in AB than in FB (the mean is 96 μm for AB vs. 76 μm for FB; *p* = 0.021). Average osteocyte lacunar density is found to be higher in AB than in FB (mean 22,874 mm^3^ vs. 19,376 mm^3^ for FB; *p* = 0.038), which might compensate for the high distance from the mineral to the nearest vessel. No significant differences in lacunar volume are found between paired AB and FB.

**Discussion:** A comparable vessel network and similar distribution of vessel porosity between AB and FB may allow the FB graft to exhibit a high regeneration potential when connected to AB, and this might correlate with a high osteoinductive and osteoconductive potential of FB when connected to AB. Since widely used and potent synthetic bone grafts exist, new insight into the bone structure of well-established autologous bone grafts, such as the free fibula flap, could help to improve the performance of such materials and therefore the design of 3D scaffolds.

## 1 Introduction

Bone healing is known to be a complex physiological process involving multiple interacting cells and signalling molecules (Vidal et al., 2020). In general, minor fractures and small bone defects heal spontaneously, whereas critical-sized and/or complex bone defects (CSBDs) still present a challenge in reconstructive surgery. Normally, CSBDs exceed the intrinsic capacity of bone healing, which might lead to delayed or insufficient bone union (Vidal et al., 2020). Defects with a length greater than 1–2 cm and a loss of more than 50% of bone circumference are generally defined as CSBDs in literature (Keating et al., 2005; Sanders et al., 2014). Animal studies demonstrated that the critical size for segmental mandibula defects in minipigs is 6 cm when the periosteum is preserved and 2 cm when the periosteum is removed (Ma et al., 2009). Since bone remodelling in pigs and humans is comparable (1.2–1.5 mm/day vs. 1.0–1.5 mm/day, respectively) (Pearce et al., 2007), these animal studies provide a valuable way to determine CSBD in humans. In oncological head and neck surgery, the required quantity of bone generally exceeds the potential of self-healing and osseous free flaps with microsurgical anastomosis are therefore often needed for reconstruction. The free fibula flap (FFF) has become the gold standard for autologous reconstruction of such defects resulting from oncological head and neck surgery (Fliss et al., 2021). Advantages of FFF are sufficient quantity of bone, possibility of ideal contouring, vascular supply, and a long pedicle (Chaine et al., 2009; López-Arcas et al., 2010; Verhelst et al., 2019; Rosen et al., 2022). Lately, by using CAD/CAM planning for FFF, a high accuracy of reconstruction and improved aesthetic and functional outcome could be achieved (Mahendru et al., 2020).

On the other hand, it is known that FFF harvesting may lead to severe donor site morbidities, which are divided into early and late complications. Early complications include infections, wound dehiscence, and delayed wound healing (Zimmermann et al., 2002; Chaine et al., 2009; Ling and Peng, 2012). Late complications comprise chronic pain, gait abnormality, limited range of motion in the ankle, and reduced sensory deficit and muscle strength, which may especially impair a patient’s quality of life (Chaine et al., 2009; Ling and Peng, 2012). This is linked to a rising demand for a synthetic three-dimensional (3D) bone scaffold with predictable anatomic traits that might replace FFF in the future. Therefore, an understanding of skeletal site-specific 3D microarchitecture as well as the physical and cellular properties of the human jaw as a recipient site and the donor bone is essential. Synchrotron micro computed-tomography (SR µ-CT) of alveolar bone (AB) and iliac crest (IC) reveals a site-specific distribution of the intracortical pore system (IPS) and osteocyte lacunar density (Rothweiler et al., 2022). On a nanostructural level, proteome-based profiling of patient-paired human AB and IC, highlights site-specific and interindividual differences (Fretwurst et al., 2022). These insights into site-specific properties may be important for the understanding of mineral homeostasis as well as mechanical and clinical performance of bone transplants regarding an osseous union with jawbones (Rothweiler et al., 2022) and may help explain the clinical success of IC in major (>5 mm) avascular bone augmentations (Nelson et al., 2006; Heberer et al., 2009; Fretwurst et al., 2015; Troeltzsch et al., 2016).

It is known that a physiological framework promotes superior ingrowth of vessels and migration of osteogenic cells, resulting in improved bone formation (Qu et al., 2021), which is seen as the main driving force for bone regeneration in nonviable and synthetic bone substitute scaffolds (Weber, 2019). Vascularization as a main factor for ideal molecular exchange and functional performance is recognized and has led to promising advances in bone tissue engineering (Sharma et al., 2019; Chen et al., 2020; Gonçalves et al., 2021). The significance of vessel orientation in 3D scaffolds was largely ignored (Sharma et al., 2019) until blood vessel supply, osteocytes, and their lacunar-canaliculi network (LCN) as well as their role in bone metabolism were recently closely examined-for example, in femoral bone (Carter et al., 2013a; Dong et al., 2014; Hesse et al., 2014; Portier et al., 2020).

It is well known that LCN plays a fundamental role in cell–cell contact, nutrition, signalling molecules, and even interactions with extracellular tissue (Hesse et al., 2015; Bortel et al., 2022). Besides the important role of LCN in mechanosensation and transduction processes of osteocytes (Burger and Klein-Nulend, 1999; Vatsa et al., 2008; Schneider et al., 2010), it has been reported that LCN is involved in bone remodelling (Currey, 2014).

To date, SR µ-CT has neither been used in a study to examine bone samples of the fibula bone (FB) as a donor, nor of the jaw bone (AB), as a recipient site in an individual. Further, it is unknown whether bone transplants for microsurgical anastomosis require specific morphological properties for the reconstruction of CSBDs or if a microarchitecture related to the recipient bone site might be favourable. Moreover, fluid simulation-derived orientation-dependent permeability properties may be of relevance, since a better match in terms of spatial orientation of the FB when connected to AB, could lead to an improved supply of nutrients and subsequently to bony union in the segmental gap. Therefore, the fluid simulation-derived permeability tensor was analysed to gain insights into the orientation-dependent permeability of AB and FB.

In order to provide a better understanding of the microarchitecture of human AB and FB, patient-paired samples were analysed and compared using SR µ-CT down to a voxel-size of 640 nm. It is demonstrated that SR µ-CT represents an ideal tool for 3D imaging of the bone microarchitecture without sample destruction. Additionally, spatial arrangement of the intracortical-pore system as well as osteocyte lacunar density and lacunar morphology were analysed and compared.

## 2 Materials and methods

The Ethics Committee of the Faculty of Medicine Charité Berlin approved this study (EA4/161/18). The study was performed in accordance with the Helsinki Declaration of 1964 as revised in 2013.

### 2.1 Patients

In total, 8 patients (females: 3; males: 5; mean age: 60.38 years; age range: 47–73 years) were treated at the Department of Oral and Maxillofacial Surgery, Charité–Universitätsmedizin Berlin, Germany and were consecutively enrolled between September 2018 and May 2020. Seven patients received a partial resection of the mandible, whereas one patient received a resection of the maxilla. A resection of the mandible or the maxilla was required due to oral squamous cell carcinoma (*n* = 6), basal cell carcinoma (*n* = 1), or osteosarcoma (*n* = 1). Primary CAD/CAM-planned reconstruction with a free fibula flap (FFF) was performed in all patients, whereby the CAD/CAM planning procedure followed a previously described workflow (Kreutzer et al., 2022). Informed consent was obtained from all participants included in this study. Patients with medication affecting bone metabolism (e.g., immunosuppressants and antiresorptive agents), were not included in this research.

### 2.2 Sample harvesting and preparation

In each patient, two bone samples were harvested (one AB and one FB sample). All samples were retrieved using an oscillating saw. The AB samples were taken from the vicinity of the connecting area of the original bone to FFF during bone contouring. Since all FFF’s were CAD/CAM-planned and raised using CAD/CAM templates, the slice of bone between two segments (which was not further used) was taken as the FB sample. Patient data are listed in Table 1.

**TABLE 1:** Patient data.

Patient	Gender	Age (years)	Sample	Biopsy origin	Diagnosis
#					
1* ^#^	Male	52	54A	Mandible region 32/33	OSCC
			54F	Fibula	
2* ^#^	Male	60	55A	Mandible region 32/33	OS
			55F	Fibula	
3* ^#^	Male	62	56A	Mandible region 33/34	OSCC
			56F	Fibula	
4*	Male	73	57A	Mandible region 43	OSCC
			57F	Fibula	
5^#^	Female	53	63A	Mandible region 34	OSCC
			63F	Fibula	
6^#^	Female	66	67A	Maxilla region 32	BCC
			67F	Fibula	
7^#^	Female	47	68A	Mandible region 34	OSCC
			68F	Fibula	
8^#^	Male	70	69A	Mandible region 44	OSCC
			69F	Fibula	
*N* = 8	62.5% male 37.5% female	Ø 60.38 years	*N* = 16	Recipient site: 87.5% mandible (*n* = 7), 12.5% maxilla (*n* = 1); Donor site: 100% fibula	Diagnosis: 75% OSCC (*n* = 6); 12.5% BCC (*n* = 1); 12.5% OS (*n* = 1)

In total, 8 patients were included. All patients’ data such as gender, diagnosis (BCC = basal cell carcinoma; OSCC = oral squamous cell carcinoma; OS = osteosarcoma), age, sample and biopsy origin are provided in this table. *patients included in the 2.27 µm series; ^#^patients included in the 640 nm series.

After harvesting, the bone samples were fixed in 4% neutral buffered formalin. Subsequently, dehydration in ascending alcohol series (water, 70%/80%/96%/100% ethanol) for three days each followed. The samples were defatted in xylene and infiltrated, embedded, and polymerized in Technovit^®^ 9100 New (Heraues Kulzer, Wehrheim, Germany). Technovit^®^ 9100 New was used according to the manufacturer’s manual (Kulzer, 2012). All Technovit^®^ blocks were cut to a size of 5 mm × 5 mm using a band saw (Proxxon S.A., Wecker, Luxembourg).

### 2.3 Synchrotron computed tomography imaging

Samples of four patients were SR µ-CT scanned at a voxel size of 2.27 µm, and samples from seven patients (including 3 that were analyzed at the 2.27 voxel-size setup) were scanned at 640 nm voxel size to address the difference in bone morphology at two different voxel-sizes [Figures for all samples are listed in Supplementary Figure S1 (2.27 µm), respectively Supplementary Figure S2 (640 nm)]. The imaging of the 640 nm data was performed at Synchrotron Soleil, France. A SR µ-CT setup of the Psiché (PSICHÉ) beamline was used with a beam energy of 45 keV and a sample to detector distance of 45 mm. For each sample, a total of 5600 projections were exposed for 40 ms each and were recorded over an angle of 360° using a Hamamatsu ORCA Flash sCMOS camera. Phase retrieval and image reconstruction was performed using Paganin’s method (Paganin et al., 2002) and a filtered back projection algorithm, respectively with a delta/beta ratio of approximately 65. Data of 2.27 µm voxel-size were acquired at the beamline ID 19 of the European Synchrotron Radiation Facility (ESRF) in Grenoble, France using 46.9 keV beam energy and a sample detector distance of 460 mm. For each sample, 4000 radiographs over an angle of 360° were recorded at an acquisition time per frame of 20 ms. A pco.edge 5.5 camera (PCO AG, Kelheim, Germany) was used. As with the 640 nm data, reconstruction was carried out using Paganin’s method in combination with the conventional filtered back projection algorithm employing a delta/beta ratio of 350 (Paganin et al., 2002).

### 2.4 Segmentation of samples

For all samples, representative volumes of interest (VOIs) were selected with a particular emphasis on artifact-free and physiological bone anatomy. In FB, cortical regions were chosen; and AB was mostly assigned to the cortical bone. The VOI of the 640 nm scan was set within the VOI scanned at 2.27 µm. Within each VOI, different tissue regions were segmented into mineralized tissue, vessel pores, and osteocyte lacunae. For both voxel-sizes (2.27 and 640 nm), inhouse-developed MATLAB (R2018b, The MathWorks Inc., Natick, MA, United States) scripts combining several different morphological operations were used. To obtain the mineral mask on the binarized data, a closing of all pores (vessel pores and osteocyte lacunae) within the mineralized region was achieved by a Euclidian distance map coupled to a thresholding step. Combining the mineral mask and the inverse of the binarized data resulted in a mask containing vessel pores. Osteocyte lacunae were extracted from the gray value data with a bottom-hat transformation and a following thresholding step. A connected component analysis was used to size and filter osteocyte lacunae between 30 and 8,000 μm^3^ and with length and width below 130 and 30 µm.

### 2.5 Digital bone morphometry and osteocyte lacunae segmentation

For all VOIs, the following parameters of bone morphometry were obtained: bone volume (BV), total volume (TV), bone surface area (SA), porosity (1-BV/TV), specific bone surface (SA/BV), osteocyte lacunae density (N.Lc/BV), mineral distance [(50%) *L*_*dist,min50*_], mineral distance [(95%) *L*_*dist,min95*_], vessel distance [(50%) *L*_*dist, vessel50*_], vessel distance [(95%) *L*_*dist, vessel95*_], and blood vessel volume (Vv.). Additionally, 640 nm scans were used to extract the shape of the individual lacunae, which were described by the ratio of the lacunae axes lengths (L_1_/L_3_; L_2_/L_3_; L_1_/L_2_, with L_1_ being the longest and L_3_ being the shortest axis). A detailed summary of the characterized morphological parameters is given in Table 2.

**TABLE 2:** All parameters extracted to characterize bone morphology (Bouxsein et al., 2010; Dempster et al., 2013).

Parameter	Symbol	Unit	Calculation/Reference	Voxel-size
Total volume	TV	mm³	Total bone volume, comprising mineral and pore volumes	2.27 µm
				640 nm
Mineral volume	BV	mm³		2.27 µm
				640 nm
Lacunar volume	LcV	µm³		2.27 µm
				640 nm
Lacunar porosity	Lc_V_/BV	%	Lc_V_/BV*100	2.27 µm
				640 nm
Lacunar density	NLc/BV	1/mm^3^		2.27 µm
				640 nm
Mineral distance (50%)	L_dist,min50_	µm	Distance maps, histogram and cumulative summation	2.27 µm
				640 nm
Mineral distance (95%)	L_dist,min95_	µm	Distance maps, histogram and cumulative summation	2.27 µm
				640 nm
Vessel distance (50%)	L_dist, vessel50_	µm	Distance maps, histogram and cumulative summation	2.27 µm
				640 nm
Vessel distance (95%)	L_dist, vessel95_	µm	Distance maps, histogram and cumulative summation (Dong et al., 2014)	2.27 µm
				640 nm
Mean value of lacunar volume	Ṽ_lac_	µm^3^		2.27 µm
				640 nm
Standard deviation of lacunar volume	σl_ac_	µm^3^		2.27 µm
				640 nm
Blood vessel volume	VV	µm³		2.27 µm
				640 nm
Vessel porosity	VV/BV	%	Vv/BV*100	2.27 µm
				640 nm
Level of alignment	LOA	-	Product of the variance of theta s_Θ_ and the variance of phi s_Φ_ (LOA = s_Φ_ ⋅ s_Θ_)	2.27 µm
Angle Phi	Φ	°	Angle between a segment and the *z*–axis. Phi = 0° if the segment is parallel to a bone´s long axis	2.27 µm
Angle Theta	Θ	°	Projected angle between a segment and the *x*–axis. The segment is parallel to a bone’s surface if theta = 0°	2.27 µm
Ratio EV mean	EV mean	-	Mean of the ratios of the eigenvectors of the permeability tensor [(E2/E1) + (E3/E1)]/2	640 nm
PrincipalAxis Length ratio L1/L3	L_1_/L_3_	-	Ratio of the longest and shortest axes of the osteocyte lacunae	640 nm
Principal Axis Length ratio L1/L2	L_1_/L_2_	-	Ratio of the longest and second-longest axes of the osteocyte lacunae L_1_/L_2_	640 nm
Principal Axis Length ratio L2/L3	L_2_/L_3_	-	Ratio of the second-longest and shortest axes of the osteocyte lacunae L_2_/L_3_	640 nm

### 2.6 Absolute permeability and tensor of absolute permeability

Fluid simulation was performed for all 2.27 µm scans. The tensor of permeability was calculated through the Absolute Permeability Tensor Calculation module (Thermo Scientific Avizo Software, XLab Module, Version 2020.2). This tensor enables the computation of permeability in all three spatial directions as well as the computation of eigenvectors and their associated eigenvalues. The ratios of three eigenvalues (E1, E2 and E3) to each other were determined to quantify the anisotropic behavior of absolute permeability (with E1>E2>E3). The termination conditions for the simulations were that either the convergence criterion was smaller than 10^−5^ or the number of iterations reached 10^6^. Tensor calculation is based on a mathematical approach which considers the sample to be representative of an infinite or macroscopic material. It provides the intrinsic permeability tensor by solving the closure problem derived from the Strokes equation by volume averaging (Whitaker, 1999). All samples were computationally aligned in the same way to ensure comparability. Therefore, the bone surface was located within the x–z plane, where the anatomical long axis was parallel to the *z*-axis of the global coordinate system.

### 2.7 Quantifying orientation of the vessel pore

Morphological skeletonization was used to extract central segments of all branches of the vessel pore network. The angles theta and phi were computed for every segment of the skeleton to allow a quantification of spatial orientation. Phi is herein defined as the angle between the segment and the *z*-axis, with phi = 0° meaning that the segment is parallel to the bone’s long axis. Theta is defined as the projected angle between the segment and the *x*-axis, resulting in a segment that is parallel to the bone’s surface if theta = 0°. Histograms showing the distribution of theta and phi for all segments in each sample were calculated and the variance of the amplitudes of phi and theta was extracted. Highly aligned segments result in heterogeneous histograms with a prominent peak and thus a high variance. In contrast, equally distributed vessel orientations show a homogeneous histogram and thus a small variance. To quantify the alignment of the vessel pores, we used a recently introduced descriptor (Bortel et al., 2022)- the level of alignment (LOA), which is defined as the product of the variance of theta s_Θ_ and the variance of phi s_Φ_ (LOA = s_Φ_ ⋅ s_Θ_).

### 2.8 Statistics

Statistical hypothesis tests (ANOVA, Kruskal–Wallis, Mann–Whitney and *t*-test) with *post hoc* testing (Bonferroni) were performed using IBM SPSS Statistics (version 25.0, IBM Corp., Armonk, NY, United States). *p*-values ≤ 0.05 were considered statistically significant.

## 3 Results

### 3.1 Comparison of bone morphometric parameters

The data collected at the ESRF at 2.27 µm voxel-size provided a larger field of view than did the scans collected at PSICHÉ at a higher spatial voxel-size and were thus used to quantify vessel morphology and lacunar density. The data collected at the 640 nm voxel-size provided a small field of view but allowed for the shape analysis of the osteocyte lacunae.

### 3.2 Bone gross morphology at 2.27 µm voxel-size

**FIGURE 1:**
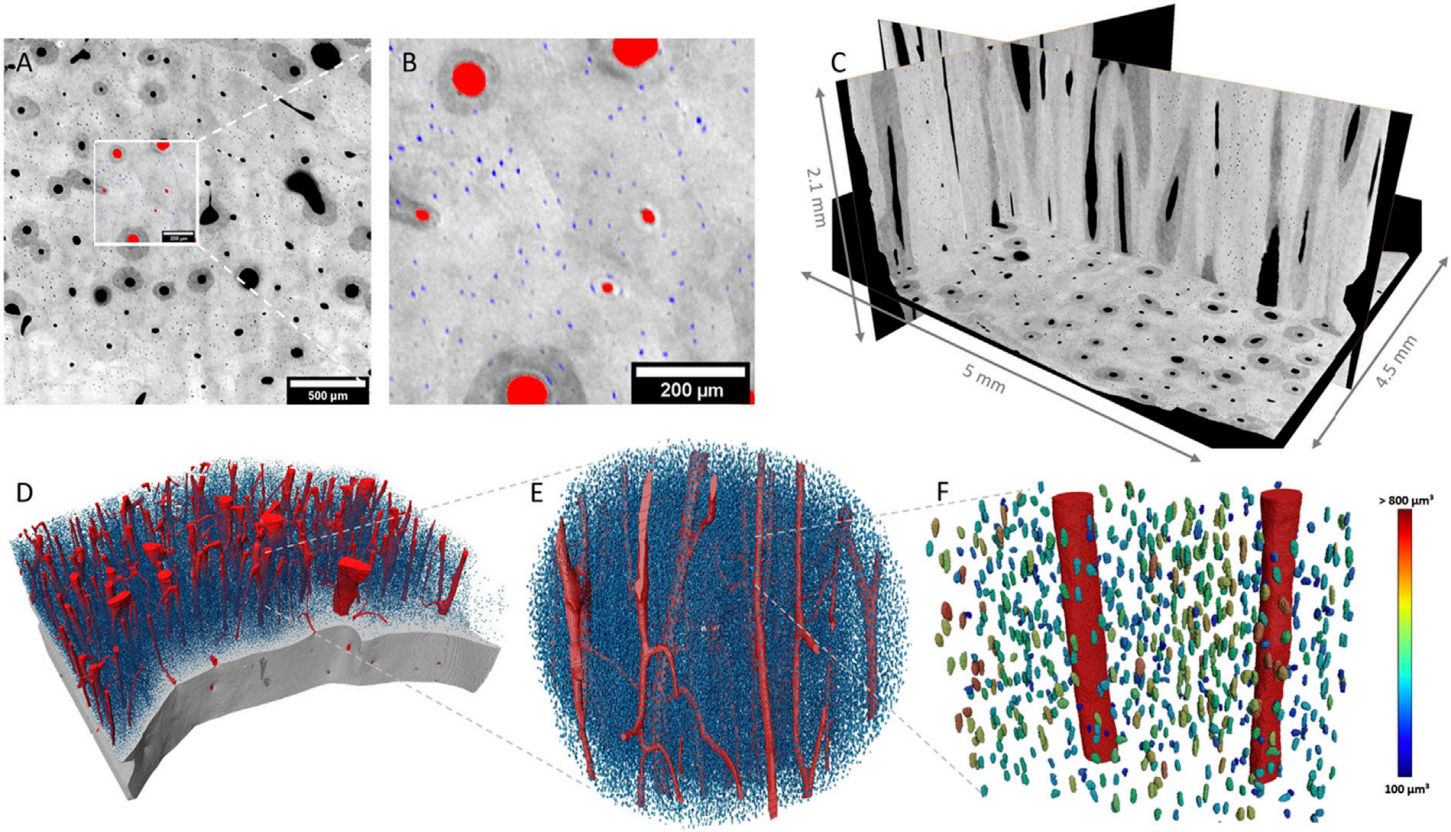
2D and 3D representation of raw and segmented data of sample 54F. Mineralized bone tissue is displayed in different shades of grey, while segmented vessels are labeled in red and lacunae in blue. **(A)** Illustrates a 2D slice inside the bone where the zoomed-in region shown in **(B)** is displayed. **(C)** Shows 3D orientation of the bone in various/selected layers. **(D)** Shows a 3D rendering of the segmented structures, where the bone is virtually cut open to reveal a vessel system and lacunae. **(E,F)** represent a selected section of D in high resolution. In **(F)**, lacunae are color-coded according to their volume.

SR µ-CT data of the 2.27 µm voxel-size scans (AB, FB and *n* = 4/each) were successfully segmented and bone gross morphology parameters were extracted as described in Table 2. Figure 1 illustrates bone sample 54F in 3D and the corresponding segmentation. In Figure 1A, it can be observed that the vessel pores in the horizontal slice mainly run perpendicular to this plane. The segmented vessels are labelled in red, while the lacunae are labelled in blue (Figures 1A, B). Orthogonal grey value slices (1C) of the bone of sample 54F are dark grey, indicating air or soft tissue compartments, while bright grey values refer to high densities of the mineralized phase. A 3D rendering of Figure 1A is given in Figure 1D: each of the blue dots refers to a single lacuna (here 18,208 mm^3^). In the zoom-in in Figure 1F, the lacunae are color-coded according to their volume.

**FIGURE 2:**
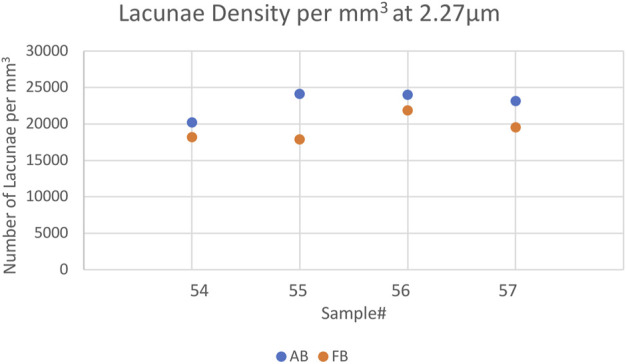
Scatterplot showing the Lacunar Density of the 2.27 µm voxelsize setup per mm^3^ for AB and FB samples 54–57. Blue indicates AB, orange represents FB.

**FIGURE 3:**
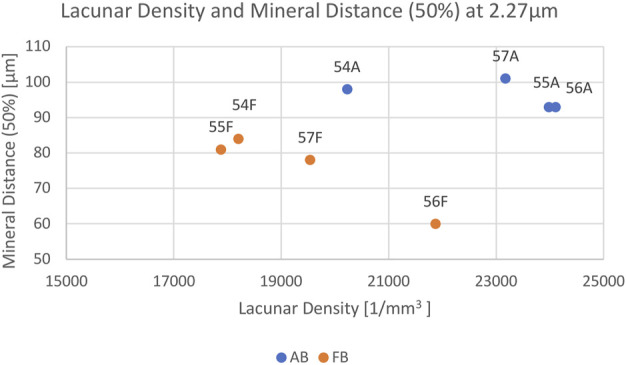
Scatterplot showing the relation between Lacunar Density per mm^3^ (*x*-axis) and Mineral Distance (50%) (*y*-axis) for AB (blue) and FB (orange) samples 54–57. Each sample is labelled.

The mean vessel porosity was found to be 8.3% for AB and 6.9% for FB. At the same time, the mean vessel distance was higher for AB than it was for FB. This was observed in the mean vessel distance of 50% (AB: 18.5 µm and FB: 13.5 µm) as well as for the mean vessel distance of 95% (AB: 71.5 µm and FB: 47 µm). On average the median distance (i.e. 50%) of the mineralized bone tissue to the closest vessel pore boundary was found to be 96 μm (minimum: 93 μm; maximum: 101 µm) for AB and 76 μm (minimum: 60 μm; maximum: 84 µm) for FB. On average, 95% of mineralized bone was found at a distance of 208 µm (minimum: 188 μm; maximum: 226 µm) for AB and 174 µm (minimum: 125 μm; maximum: 196 µm) for FB from the canal surface. The average lacunar density for AB was found to be 22,874 per mm^3^ (minimum: 20,228; maximum: 24,108) and 19,376 per mm^3^ (minimum: 17,882; maximum: 21,873) for FB. A summary of the results is given in Table 3 and a visualization of statistically significant results is provided in Figures 2, 3 (and Supplementary Figure S3).

**TABLE 3:** Bone morphological parameters extracted for AB and FB samples at a voxel-size of 2.27 µm.

Sample	Vessel porosity (%)	Lacunar density (per mm^3^)	Mineral distance (50%) (μm)	Mineral distance (95%) (μm)	Vessel distance (50%) (μm)	Mineral volume in mm^3^	Vessel distance (95%) (μm)	Vessel Surface/Vessel volume (1/mm)	Vessel Surface/Total volume (1/mm)	Vessel Surface/Bone volume (1/mm)
54A	15.82	20,228	98	226	29	17.5	120	22.8	2.6	2.9
54F	3.82	18,208	84	196	11	28.9	42	53.6	2.0	2.1
55A	3.65	24,108	93	205	10	3.7	43	60.5	2.2	2.3
55F	5.84	17,882	81	195	11	15.2	37	52.5	3.1	3.3
56A	3.22	23,984	93	188	9	13.5	31	59.7	1.9	2.0
56F	6.38	21,873	60	125	10	18.0	33	57.2	3.6	3.9
57A	10.63	23,175	101	213	26	25.3	92	23.1	2.5	2.8
57F	11.62	19,541	78	179	22	21.8	76	28.9	3.4	3.8
t-test p =	Not significant	**0.038**	**0.021**	Not significant	Not significant	Not significant	Not significant	Not significant	Not significant	Not significant

The bold values provide statistically significant values.

Most parameters showed no significant differences between AB and FB except for lacunar density (*p* = 0.038) and mineral distance (50%) (*p* = 0.021).

### 3.3 Lacunae shape descriptors at 640 nm

As described, gross bone morphology parameters were also determined for 7 patient-paired bone samples at a voxel-size of 640 nm (Table 4). Mean lacunar density per mm^3^ showed the same trend and was higher for AB (25,145; ranging from 20,978 to 28,416) than it was for FB (21,042; ranging from 17,807 to 23,068), thus revealing significant differences between these bone entities (*p* = 0.0049). Mean vessel porosity for both bone entities was comparable: it was 5.16% (1.03%–11.27%) for AB and 5.61% (2.91%–8.48%) for FB. For the 640 nm series, lacunae shape descriptors were additionally characterized. The mean volume of the osteocyte lacunae was found to be slightly lower in AB with a mean volume of 553 μm^3^ (from 303 to 851 μm^3^) than it was in FB with a mean volume of 601 μm^3^ (466–698 μm^3^). Principal axis length ratios were measured and compared. In AB, the mean L1/L3 and L1/L2 was found to be slightly higher (3.0 and 2.3, respectively) than it was in FB (2.9 and 2.1, respectively). Mean L2/L3 was 1.37 for AB and 1.41 for FB. Interestingly, no statistical difference in the volume of the lacunae could be found between AB and FB. Sample 67 A—the only AB harvested from the maxilla showed similar results for the lacunar shape descriptors (Table 4).

**TABLE 4:** Lacunar shape descriptors for AB and FB samples examined at 640 nm.

Sample	Number of lacunae	Mineral volume in mm^3^	Lacunar density (per mm^3^)	Mean value of the lacunar volume ± SD (μm^3^)	Standard deviation of lacunar volume (μm^3^)	Vessel porosity (%)	Principial axis length ratio L1/L3 ± SD (-)	Principial axis length ratio L1/L2 ± SD (-)	Principial axis length ratio L2/L3 ± SD (-)
54A	55,543	2.65	20,978	627 ± 295	295	11.27	2.9 ± 0.7	2.2 ± 0.7	1.3 ± 0.2
54F	85,134	4.40	19,354	698 ± 280	280	2.91	3.2 ± 0.8	2.2 ± 0.7	1.5 ± 0.3
55A	33,269	1.21	27,470	414 ± 183	183	2.07	2.8 ± 0.9	2.1 ± 0.7	1.4 ± 0.3
55F	48,039	2.33	20,625	657 ± 268	268	3.63	3.1 ± 1.0	2.1 ± 0.7	1.5 ± 0.3
56A	59,501	2.37	25,136	303 ± 145	145	3.41	3.4 ± 1.2	2.6 ± 1.0	1.4 ± 0.2
56F	14,956	0.71	20,975	604 ± 306	436	6.90	2.7 ± 0.8	2.0 ± 0.7	1.4 ± 0.2
63A	16,886	0.71	23,860	559 ± 254	254	1.07	3.2 ± 0.9	2.4 ± 0.8	1.4 ± 0.3
63F	32,253	1.39	23,279	541 ± 237	237	4.87	2.8 ± 0.7	2.2 ± 0.7	1.3 ± 0.2
67A*	10,706	0.42	25,647	431 ± 174	174	9.18	2.8 ± 0.8	2.2 ± 0.7	1.4 ± 0.2
67F	35,523	1.54	23,068	555 ± 251	251	7.24	2.9 ± 0.8	2.2 ± 0.7	1.3 ± 0.2
68A	59,441	2.09	28,416	851 ± 325	325	1.03	3.1 ± 0.8	2.4 ± 0.8	1.3 ± 0.2
68F	22,342	1.01	22,186	466 ± 185	185	5.21	2.7 ± 0.7	2.0 ± 0.7	1.4 ± 0.3
69A	36,837	1.50	24,508	685 ± 315	315	8.11	3.1 ± 0.8	2.3 ± 0.8	1.4 ± 0.3
69F	32,708	1.84	17,807	688 ± 299	299	8.48	3.0 ± 0.8	2.1 ± 0.7	1.5 ± 0.3
*p*-value	0.9881	Not significant	**0.004878**	Not significant	Not significant	Not significant	Not significant	**0.02946**	Not significant
*p*-value paired	0.9880	Not significant	**0.005549**	Not significant	Not significant	Not significant	Not significant	Not significant	Not significant

*Only sample AB was taken from the maxilla.

The bold values provide statistically significant values.

### 3.4 Impact of voxel-size

For 6 sample scans (54 A, 54 F, 55 A, 55 F, 56 A and 56 F), lacunar densities were compared between two different voxel-sizes (2.27 and 0.64 µm) to assess the impact of field of view positioning and the voxel-size on the lacunar density (Table 5). We found that in 5 out of 6 scans (54 A, 54 F, 55 A, 55 F and 56 A), lacunar density was found to be slightly higher for the high resolution data (Table 5) suggesting that not all lacunae were captured by the 2.27 µm voxel-size setup. For the one scan with the smaller lacunar density, the VOI of the 0.64 µm-setup was the smallest among the compared specimens and could therefore result in being not representative for the computation of the lacunar density and that the selection of a different VOI would likely result in a higher lacunar density.

**TABLE 5:** Comparison between the number of lacunae when extracted from 2.27 µm data and 640 nm data.

Sample	N.Lc/mm³ of the 2.27 µm data	N.Lc/mm³ of the 0.6 µm data	Ratio of densities between both voxel-size data
54A	20,228	20,978	1.037
54F	18,208	19,354	1.063
55A	24,108	27,470	1.139
55F	17,882	20,625	1.153
56A	23,984	25,136	1.048
56F	21,873	20,975	0.959

Sample 56F is the only sample with a high number of lacunae in the 2.27 µm scan.

### 3.5 Orientation of vessel network

**FIGURE 4:**
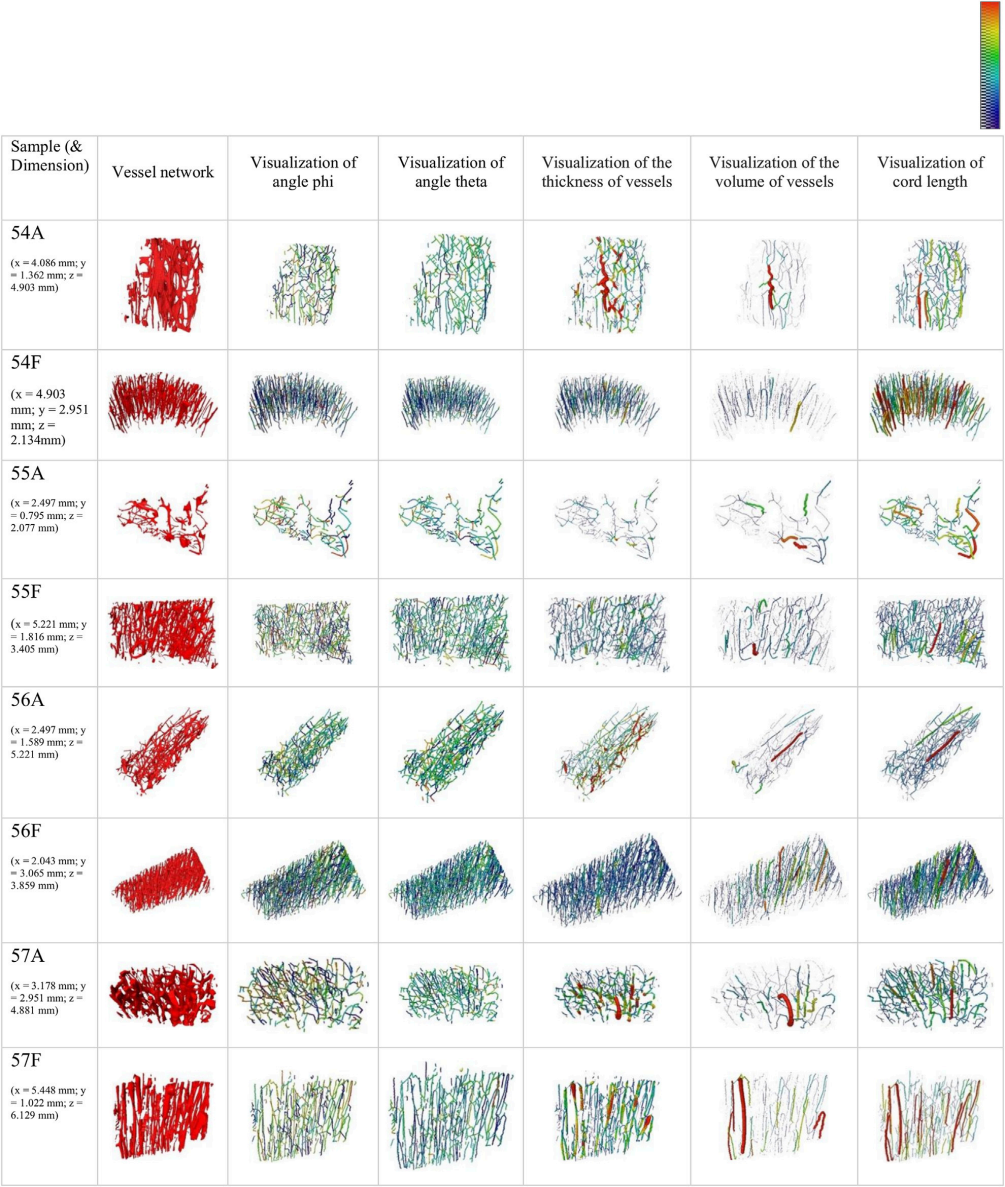
3D rendering of the segmented vessel network of 2.27 µm samples and visualization of skeletons color-coded by angles phi (colour bar: 0°–360°) and theta (colour bar 0°–90°) (column 2–4). Additionally, a visualization of pore network skeletons color-coded and weighed by thickness (colour bar: 0–30 μm) and volume (colour bar: 0 to 5 ⋅ 10^6^ μm^3^) and cord length (colour bar: 0–900 μm) (column 5–7) are shown. The thicker the vessels displayed, the larger the respective parameter.

The vessel network of AB and FB samples and the angles theta and phi as well as the visualization of the thickness, the volume of the vessels and the cord length are visualized in Figure 4. The skeleton branches are color-coded by angle, and it appears that FB samples have a high degree of alignment.

The summarized results for the orientation parameters normalized to vessel length (vessel volume) are presented in Table 6. The ratio LOA_FB/LOA_AB is smaller than 1 for samples 54 and 55 and is notable above 1 for samples 56 and 57.

**TABLE 6:** Orientation parameters normalised to the vessel length of the 2.27 µm series.

Sample	Phi bin var (E-03)	Theta bin var (E-03)	LOA (E-05)	LOA ratio (FB/AB)
54A	5.17	10.29	5.32	0.13
54F	1.03	6.73	0.70	
55A	3.98	6.32	2.51	0.61
55F	2.85	5.38	1.53	
56A	1.12	5.49	0.62	2.38
56F	2.68	5.50	1.47	
57A	1.19	8.47	1.01	2.71
57F	4.82	5.68	2.74	

Histograms of the pooled angles for phi and theta, normalized to segment length and volume, indicate a pronounced intensity between ∼35° and ∼55° (theta) for AB. Histograms for phi show pronounced peaks between 40° and 50° for FB.

Fluid simulations were computed for 4 paired samples, and results are listed in Table 7. Eigenvalues were computed and the means of E2/E1 and E3/E1 were calculated, revealing a more uni-directional pronounced permeability in FB than in AB in 3 of the 4 paired samples. The inverse of the mean ratio EV for each sample was calculated to highlight the consistent scale of paired AB and FB (Table 7).

**TABLE 7:** Results of fluid simulation for samples 54, 55, 56 and 57 showing the mean ratio of eigenvalues for each sample.

Sample	Ratio EV mean	Invert
54A	0.0037	273.4
54F	0.0024	425.5
55A	0.0768	13.0
55F	0.0171	58.5
56A	0.0162	61.7
56F	0.0338	29.6
57A	0.1509	6.6
57F	0.1237	8.1

Mean eigenvalues (with E1 > E2 > E3) of the permeability tensor and their ratio have already been computed. To illustrate, the invert number 273.4 means that the main permeability along the main permeability direction is 273.4 times more pronounced than it is in the orthogonal directions.

## 4 Discussion

This study examined patient-paired AB and FB at different length scales to quantify differences in bone morphology, including permeability properties, to comprehend factors that could be relevant to the success of microvascular free fibula bone transplants in reconstructive surgery. SR µ-CT evaluation of geometrical properties of human bone has been described before and used successfully (Hesse et al., 2014). However, to the best of our knowledge, a comparison of AB and FB on a micro scale in 3D has not been performed to date.

### 4.1 Lacunar density and morphology

Osteocyte lacunar properties in AB, tibial bone (TB) and femoral bone (FeB) have been described before, especially regarding either a variation in osteocyte lacunar density, volume, morphology, orientation or even age-dependent changes within a femur (Carter et al., 2013b; Ashique et al., 2017). In the present study, osteocyte lacunar density was found to be significantly higher in AB compared to FB. The high number of lacunae per mm^3^ found in AB, 20,228–24,108 (2.27 µm data set) and 20,978–28,416 (640 nm data set) are in accordance with previous SR µ-CT studies (Hesse et al., 2014a; Hesse et al., 2014b). Although various papers (Gauthier et al., 2018; Milovanovic and Busse, 2019) have addressed the question of inter-site variability of the osteocyte lacunar network, available data are still not fully conclusive.

Since the FB in our study was consequently standardized and harvested in the middle of diaphysis, no regional differences should occur due to different sampling locations. Gauthier et al. (2018), examined femoral diaphyses, femoral neck, and radial diaphyses and found a significantly higher lacunar density in the femoral neck compared to the radius (26,123/mm^3^ vs. 21,191/mm^3^). In contrast, the lacunar density of femoral diaphyses versus radial diaphyses showed merely slight differences (21,617/mm^3^ vs. 21,191/mm^3^) (Gauthier et al., 2018). These findings suggest that inter-site differences in one individual exist and that even anatomical regions near each other show differences in osteocyte lacunar number, which could suggest changes in bone microstructure between weight-bearing and non- or low-weight bearing areas (Gauthier et al., 2018; Milovanovic and Busse, 2019). The lacunar density of FB found in our study (17,882–21,873/mm^3^ for the 2.27 µm data set and 17,807–23,860/mm^3^ for the 640 nm data set) resembles the result for radial diaphyses as well as that for femoral diaphyses. Anatomically, radius and FB are comparable in terms of mechanical loading as both are low weight-bearing bones. We report that in 5 out of 6 scans, lacunar density was found to be higher for the 640 nm voxel-size data, suggesting that small lacunae were not identified when a large voxel setup was used. In only one sample does the 640 nm voxel-size data reveal a lower lacunar density, which might have originated from the heterogeneity within the individual sample. It must be noted that the analysed VOI of this particular sample was much smaller than that of the other samples. This implies that at 2.27 µm voxel-size of the used imaging setup and analysis, potentially undersegments about 5%–15% of the lacunae. Additionally, the shape of each lacuna was examined: the ratios of the lacunae principal axes lengths indicate a similar shape in the lacunae of AB and FB. The shape of the osteocyte lacunae and the potential influence on the function of osteocytes as a “mechanosensor” of the bone are assumed to be affected by mechanical loading of the bone (van Oers et al., 2015). Round osteocytes are considered to be more mechanosensitive compared to elongated osteocytes in long bones that are accustomed to high mechanical loadings (Bacabac et al., 2008). According to the assumption that variation in lacunar as well as osteocyte shape directly affects osteocytic mechanosensation and subsequently bone remodelling (van Oers et al., 2015), the findings of our study, which has a similar lacunar shape of AB and FB, might indicate comparable mechanosensitive properties. Wu et al. (2018), discovered significant differences in osteocyte surface area and orientation locally in the maxillary bone where the osteocyte surface area was 1.5 times higher and osteocyte-orientation was more cranially caudally orientated in tooth gaps compared to its orientation in edentulous parts of the maxilla, which may be related to tensile strain magnitude and orientation. Unfortunately, our data do not allow any conclusions to be drawn regarding local differences within an individual since only one AB and FB sample were examined per patient due to ethical and harvesting limitations. After transplantation, FB weight-bearing probably differs from its previous weight-bearing: dental implants are inserted when dental/prosthetic rehabilitation is done. Iezzi et al. (2020), found a higher number of osteocytes in the jawbone around immediately loaded implants compared to their number in unloaded bone sites, which suggests a change in bone matrix after dental implant loading. Changes in lacunar density of FB before and after dental implant insertion as well as after implant loading might be of particular interest. In addition, van Tol et al. (2020), showed that not only mechanical loading but also LCN architecture should be considered as a key determinant of bone adaption, which clears the way for further research. Given these preconditions, investigations of FB after dental implant insertion and weight-loading, due to prosthetic rehabilitation that affects this weight loading of the FB/neo-mandible, could help create an understanding of the process of adaption of the bone and the LCN to changes in mechanical loading.

### 4.2 Spatial mineral density distribution

In the present study, the mineral distance to the closest pore surface (considering 50% of all mineral volume) was found to be significantly higher in AB compared to FB, and the mineral distance (considering 95% of all mineral volume) tended to be higher in AB than in FB. Our study suggests that spatial mineral distribution is organized such that the distance to the closest vessel pore boundary is larger in AB than in FB. In contrast, AB shows a higher lacunar density than does FB, which might compensate for the great distance to the closest vessel. Recent studies have demonstrated the relation between LCN and mineralization (Roschger et al., 2019; Ayoubi et al., 2020), whereby a dense LCN is normally accompanied by a high degree of mineralization. The degree of mineralization could not be quantified in our study due to the different geometrics of the samples and the local-tomography imaging approach. The potential role of mineral exchange at the LCN interface has previously been reported in several researches (Qing et al., 2012; Kerschnitzki et al., 2013; Hesse et al., 2014a; Roschger et al., 2019).

### 4.3 Vessel network, porosity and permeability

The present results revealed an average vessel porosity (for the 2.27 µm samples) of 8.3% for AB and 6.9% for FB as well as 5.16% for AB and 5.61% for FB (for the 640 nm samples), which is consistent with the findings of previous studies (Feik et al., 1997; Bousson et al., 2000; Bousson et al., 2001; Kingsmill et al., 2007) and suggests similar characteristics in AB and FB. The average vascular porosity of different regions of the femur (midshaft, middiaphyseal, and midfemoral cortex) was found to be 7%–10% (Feik et al., 1997; Bousson et al., 2000; Bousson et al., 2001) and 3%–11% in the mandible (Kingsmill et al., 2007). Revascularization is described as the prime criterion for success in the regeneration of autologous bone grafts and transplants as well as for immobility and infection-free healing (Nelson et al., 2006). In order for a critical bone defect to heal, a newly formed bone requires a high vascularization (Lovett et al., 2009), which can be transferred to the osteotomy gap and the gap between FB and AB (as the recipient site). In maxillofacial surgery, the osteosynthesis normally needs to be removed (at least partially) prior to dental implant insertion (Kreutzer et al., 2022). Therefore, a sufficient osteogenesis with ossification of the osseous gap (FB and FB and AB and FB) is necessary. The rate of union in FFF is reported to be high (up to 97% after five years) (Houdek et al., 2017), which can be explained by the high number of vessels that follow the main axis of the graft and therefore allow a high blood supply leading to nutrient perfusion and mass transport. Additionally, in this area, the flow dynamic into the bone graft is often predefined by the recipient site/residual bone, where the orientation of the vessel network in FB might allow a high number of new vessel connections and a high number of vessels that can sprout into the osseous gap between AB and FB. The high and comparable number of vessels as well as the comparable vessel network between AB and FB may allow the FB graft to exhibit a high regeneration potential when connected to AB. This might correlate with a high osteoinductive and osteoconductive potential of FB when connected to AB, which is reflected in the similar distributions of vessel porosity.

Fluid simulation for most paired samples (three out of four) indicates a more pronounced relative permeability along the *z*-direction for FB compared to that in AB, which might correspond with the degree of osteoconductivity of FB. Unfortunately, only limited data on fluid flow simulation in patient-paired human bone samples exist (Rothweiler et al., 2022). Further studies on this simulation should be done, which could lead to an enhanced understanding of bone transplants if the composition of vessel pores and permeability becomes known. As aforementioned, a simplified approach with a single phase-flow was utilized (Rothweiler et al., 2022). This approach could be modified to create advanced models in subsequent studies.

A comparable SR µ-CT study (Rothweiler et al., 2022) to the current one focused on IC, which is an autologous bone graft known for its high clinical success in bone augmentation in oral and maxillofacial surgery (Nelson et al., 2006; Heberer et al., 2009; Fretwurst et al., 2015; Troeltzsch et al., 2016). Rothweiler et al. (2022), compared the bone morphology of AB (as a recipient/local bone) and IC. They found significantly more lacunae in AB compared to IC, which matches our findings for AB and FB. Moreover, AB showed a long distance for 50% and 95% of mineralized bone tissue to the closest vessel pore boundary (Rothweiler et al., 2022), which is in line with our results concerning mineral distance. This allows speculation that an increased number of osteocyte lacunae might compensate for the high average distance of the mineralized bone tissue to the nearest vessel in AB. Regarding the two different bone sites, IC and FB, AB evidently features different bone morphological characteristics that must be considered in designing 3D scaffolds for the maxilla and especially the mandible.

### 4.4 Limitations

The major limitation of this study is that only eight patients were included in this study, whereby samples of four patients were scanned at 2.27 µm and samples of seven patients were scanned at 640 nm voxel-size (including three patients from the 2.27 µm setup). Additionally, the samples of AB were not taken from identical regions of the mandible and one AB sample was taken from the maxilla. The segmentation of the lacunae is sensitive to the quality of the reconstructed SR µ-CT data. In turn, image quality can be a function of sample size, which also varied in different scans. Segmentation routines that are automatically adapted for each individual lacuna should be implemented for future studies. Additionally, the used voxel-size and the actual resolution must not be confused, since the resolution depends not only on the voxel-size but also on factors such as the Signal-to-Noise Ratio.

## 5 Conclusion

Vessel porosity, vessel network, vessel distance, as well as lacunar volume were not significantly different between AB and FB. Significant differences could be found for lacunar density and the average mineral-pore distance. Similarities in vessel orientation, porosity and vessel network might be beneficial to the osseous union of FFF and the jaw in reconstructive oral and maxillofacial surgeries. If differing lacunar density and average mineral-pore distance adversely affect the success of a bone graft/transplant, further research should be done. Additional studies on possible changes in a recipient site and the bone graft/transplant itself with regard to possible adaption mechanisms might help to create an understanding of how FB or AB contributes to the success of a transplant.

## Data Availability

The original contributions presented in the study are included in the article/Supplementary Material, further inquiries can be directed to the corresponding author.

## Supplementary material

SUPPLEMENTARY FIGURE S1 For each sample of the 2.27 µm voxel-size setup (54–57 A and F), SR-µCT images from all 3 angles (YZ; XZ:XY), the VOI, and a 3D rendering are listed. The corresponding scalebar is provided within each figure. A indicates alveoalar bone, and F fibula bone.

SUPPLEMENTARY FIGURE S2 For each sample of the 640 nm voxel-size setup (54–56 A and F, respectively 63, 67-69 A and F), SR-µCT images from all 3 angles (YZ; XZ:XY), as well as the VOI are listed. The corresponding scalebar is provided within each figure. A indicates alveoalar bone, and F fibula bone.

SUPPLEMENTARY FIGURE S3 Scatterplot showing the relation between Lacunar Density per mm^3^ (x-axis) and Mineral Distance (95%) y-axis for AB and FB samples 54–57. Blue indicates AB, orange represents FB.

## Abbreviations

3D, three-dimensional; AB, alveolar bone; BV, bone volume; CAD/CAM, critical-sized and/or complex bone; CSBD, critical-sized bone defect; CT, computed tomography; IC, iliac crest; IPS, intracortical pore system; LCN, osteocyte lacunar–canalicular system; LOA, level of alignment; N.Lc, number of lacunae; SA, surface area; SR µ-CT, synchrotron radiation micro-computed tomography; Tb.Sp, trabecular separation; Tb.Th, trabecular thickness; TV, total volume; VXL, voxel; VOI, volume of interest.
