# Supplementary material for: Comparison of the 3D-microstructure of human alveolar and fibula bone in microvascular autologous bone transplantation: a synchrotron radiation μ-CT study
Source: Front Bioeng Biotechnol. 2023 Aug 25;11:1169385. doi: 10.3389/fbioe.2023.1169385 (PMC10486015; doi:10.3389/fbioe.2023.1169385)

Supplementary Figure 1

S54A – 2.27 µm

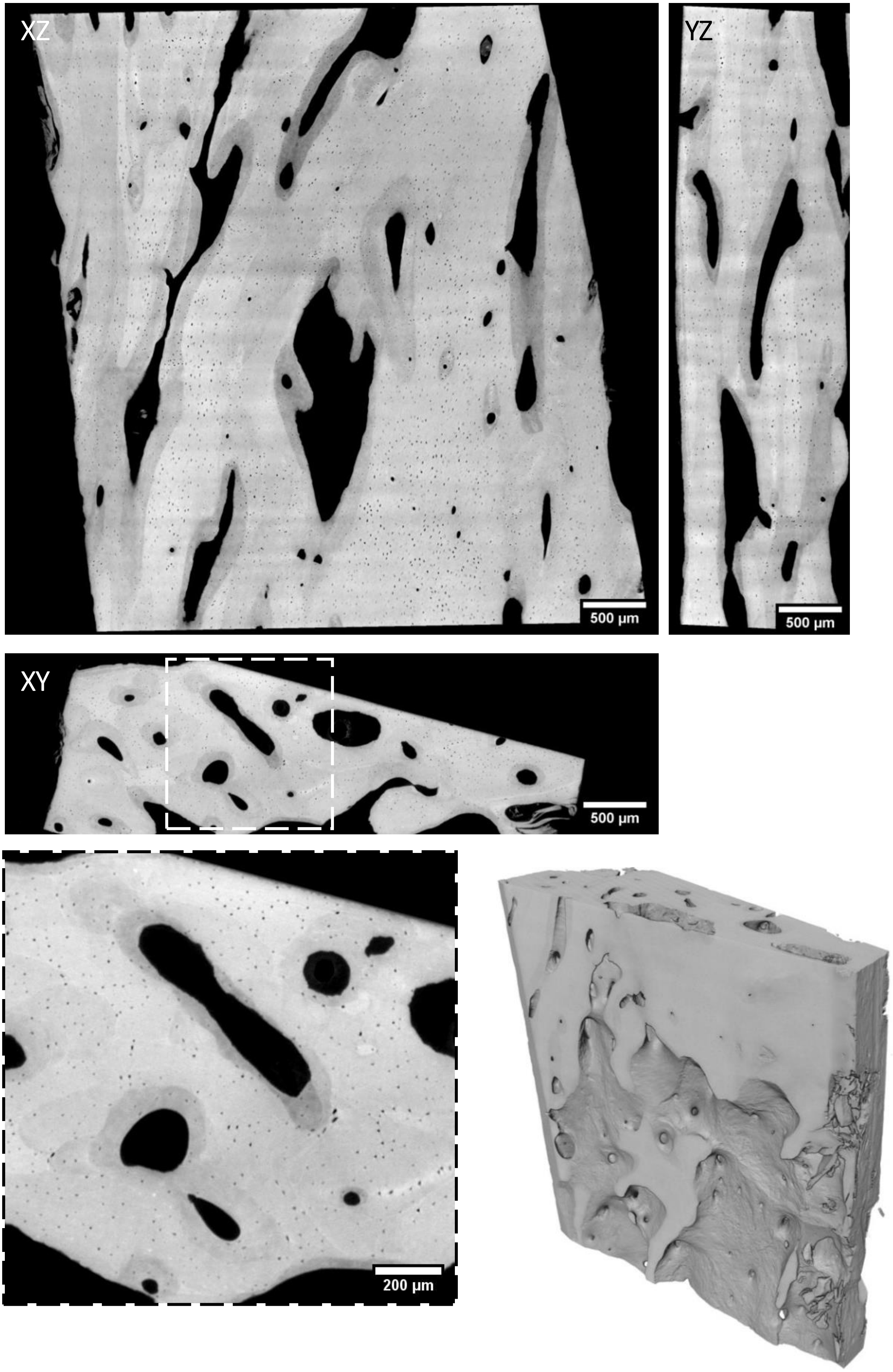

S54F – 2.27 µm

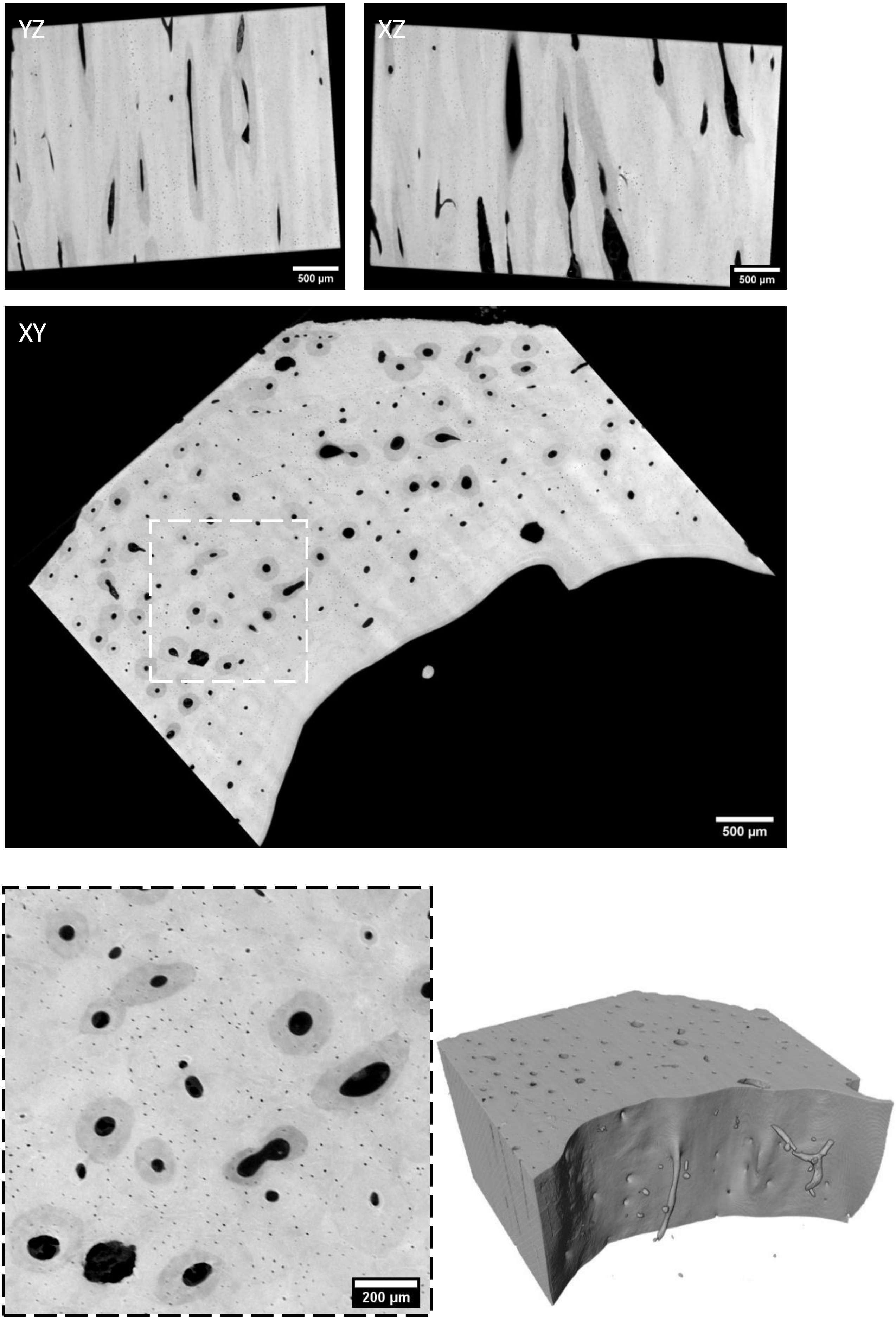

S55A – 2.27 µm

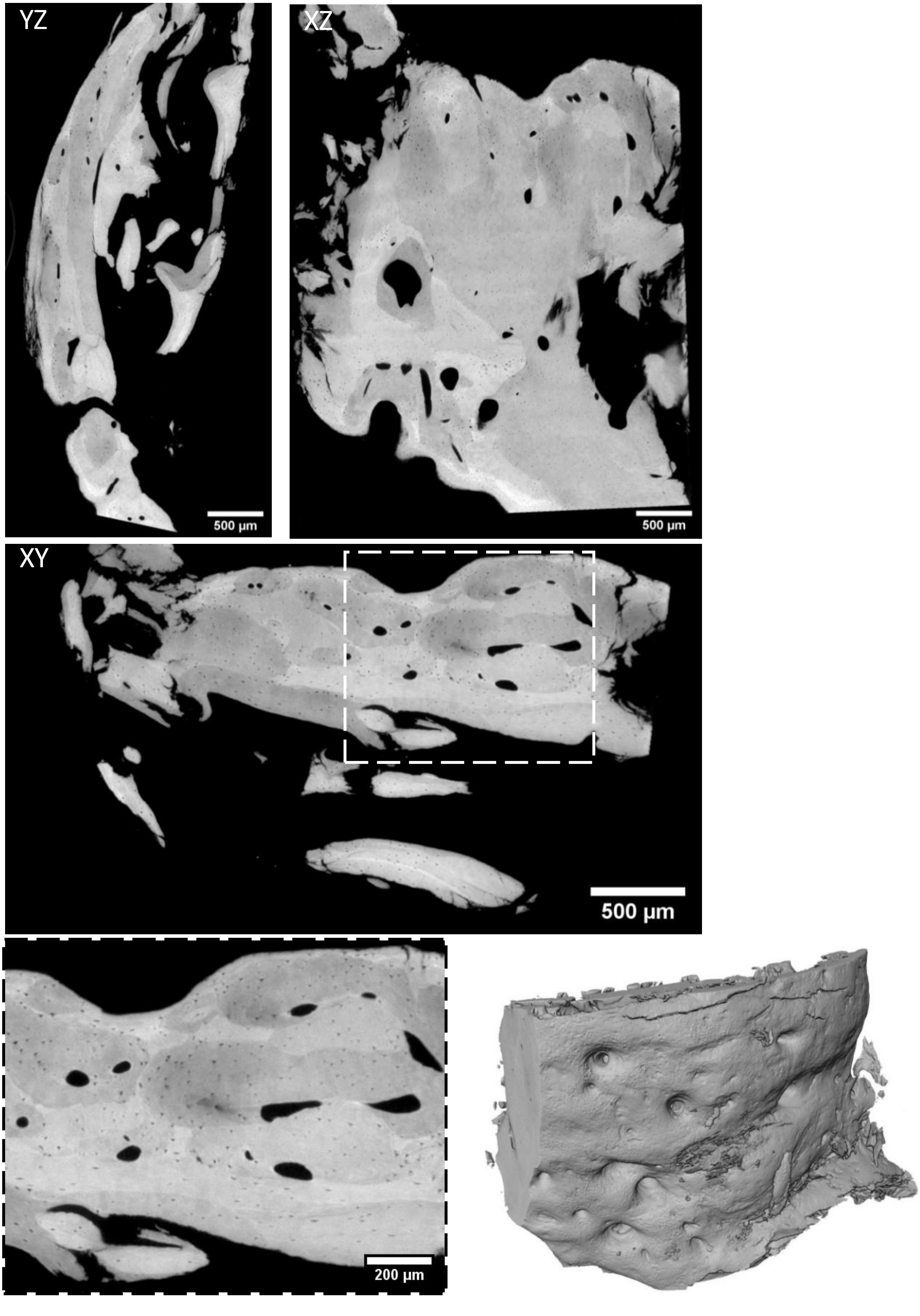

S55F – 2.27 µm

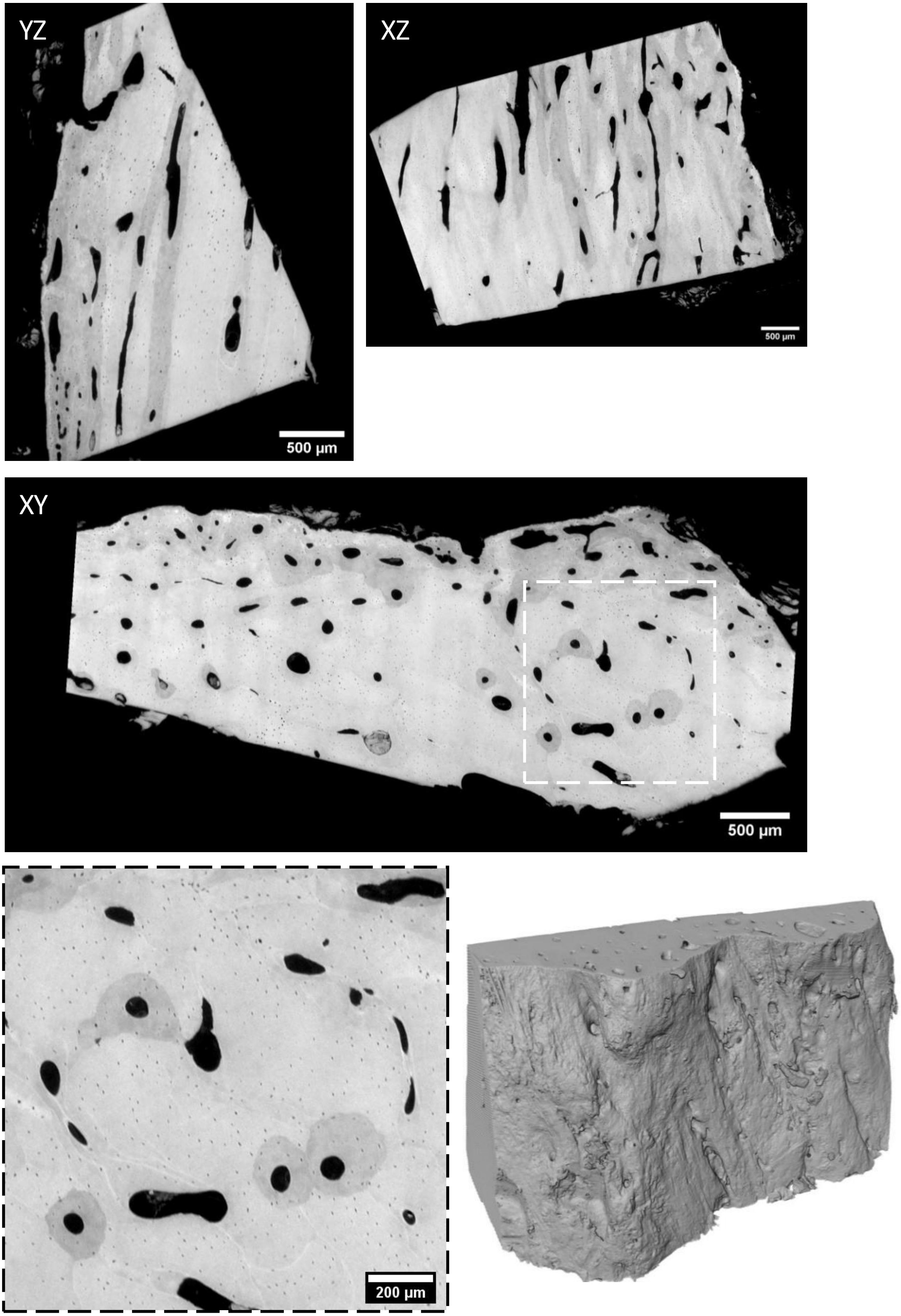

S56A – 2.27 µm

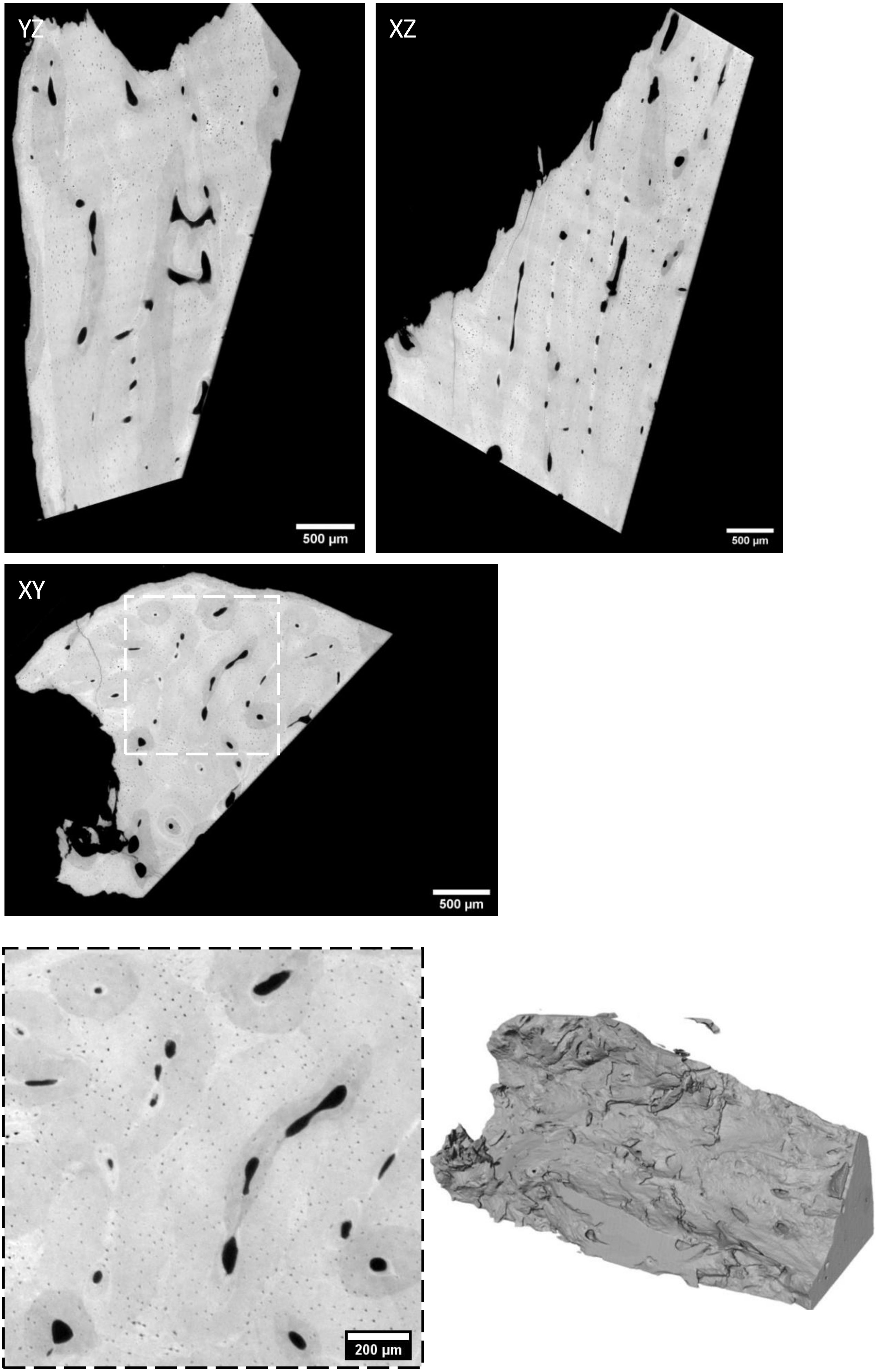

S56F – 2.27 µm

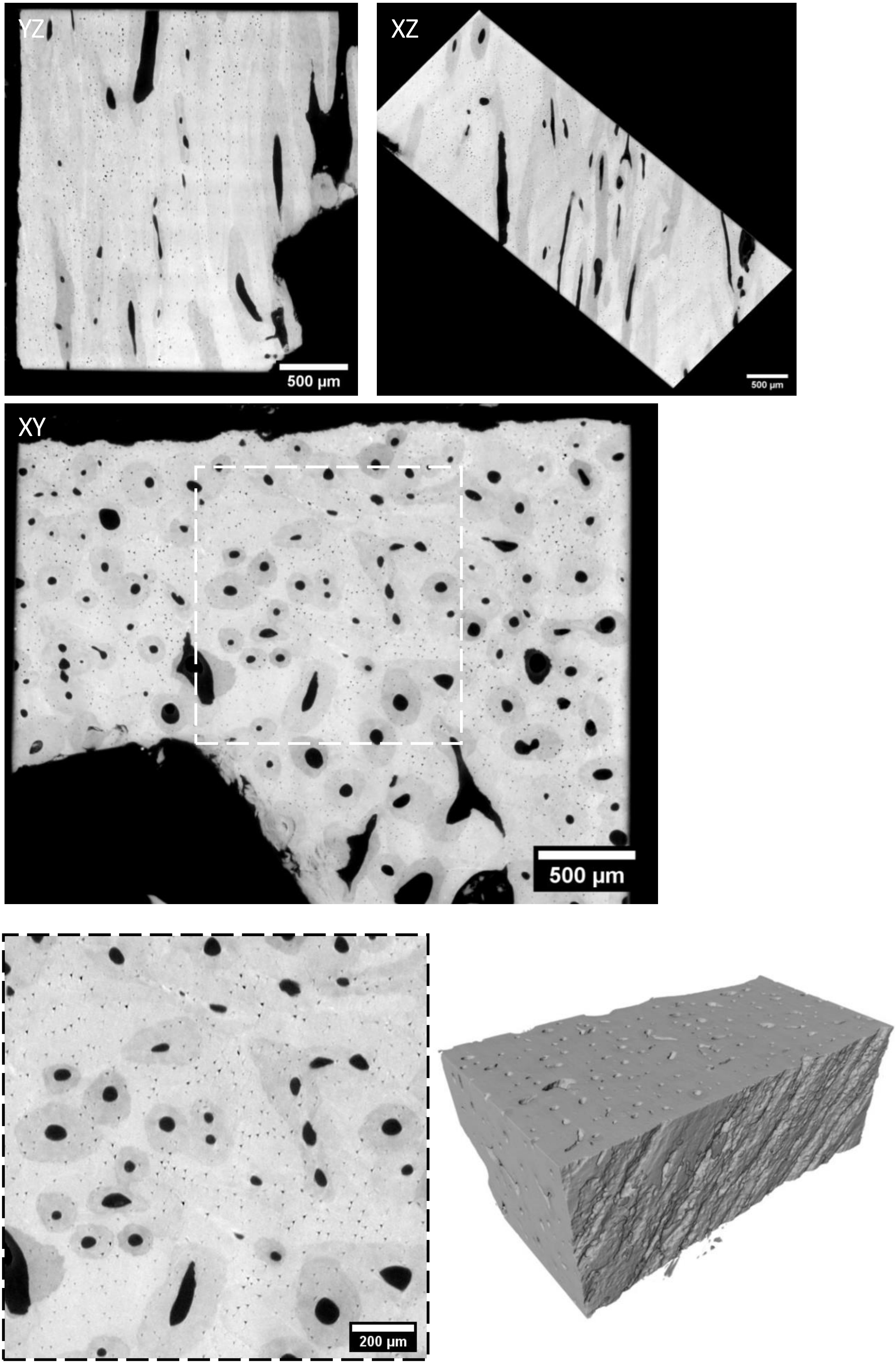

S57A – 2.27 µm

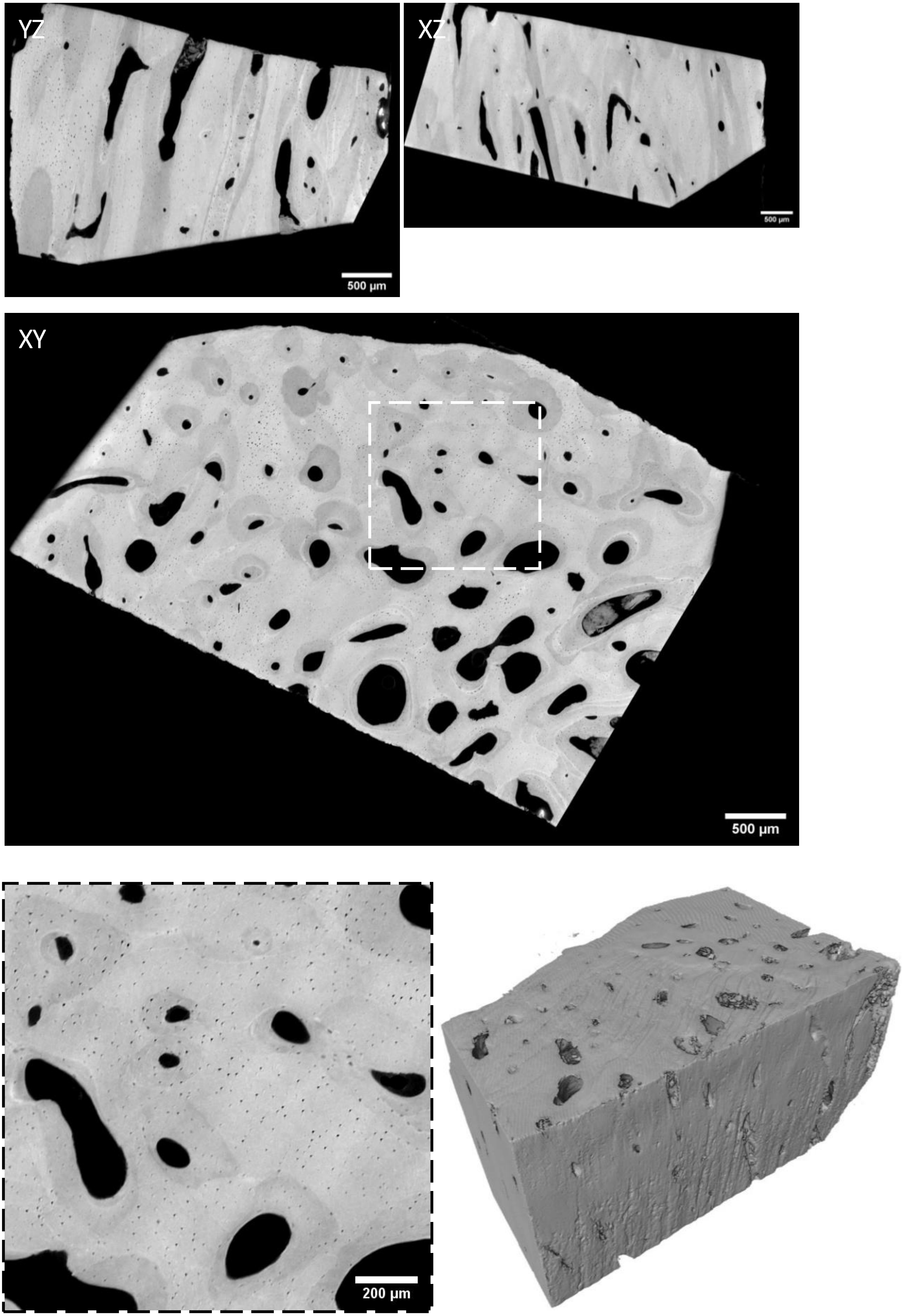

S57F – 2.27 µm

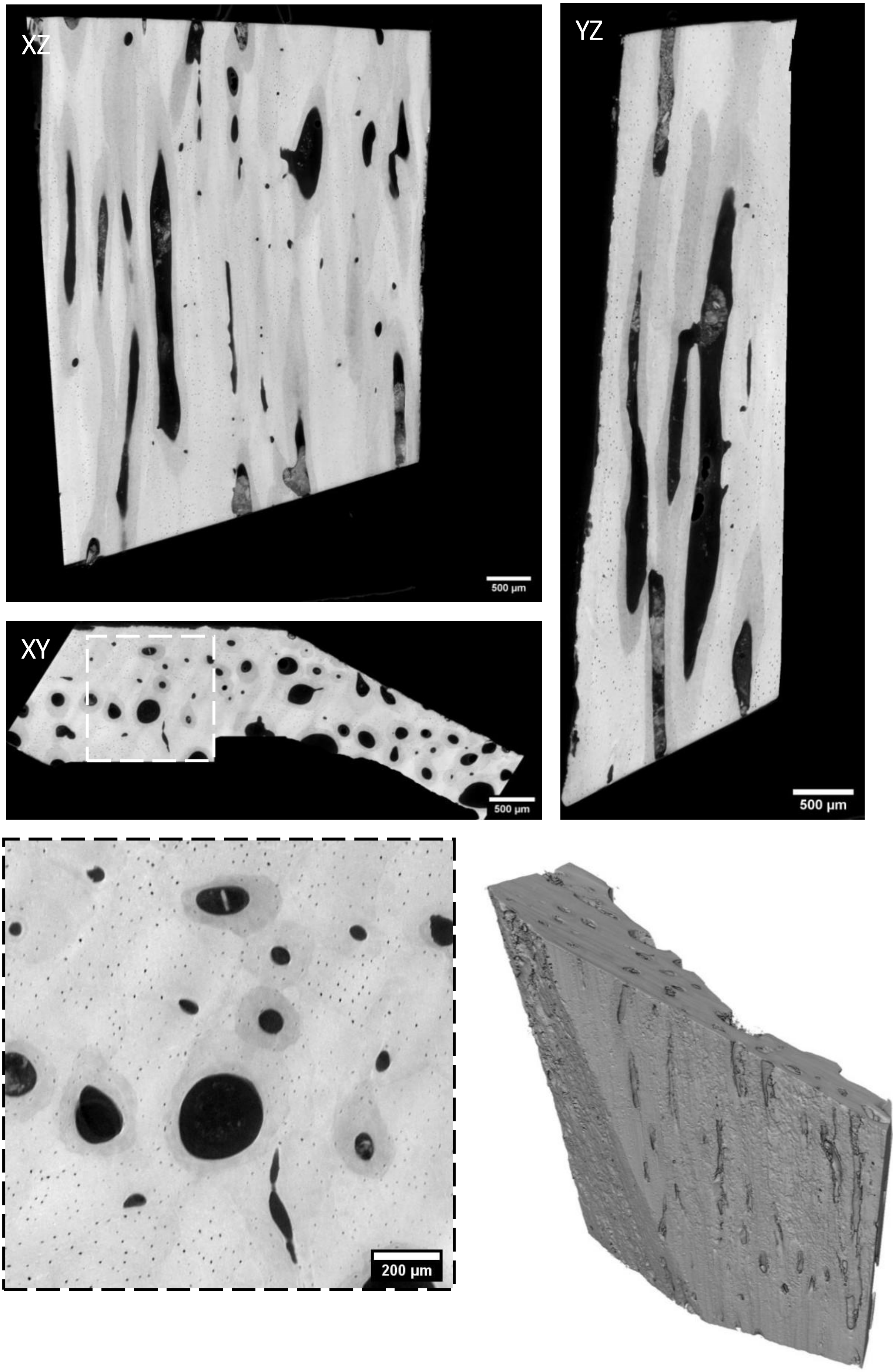

Supplementary Figure 2

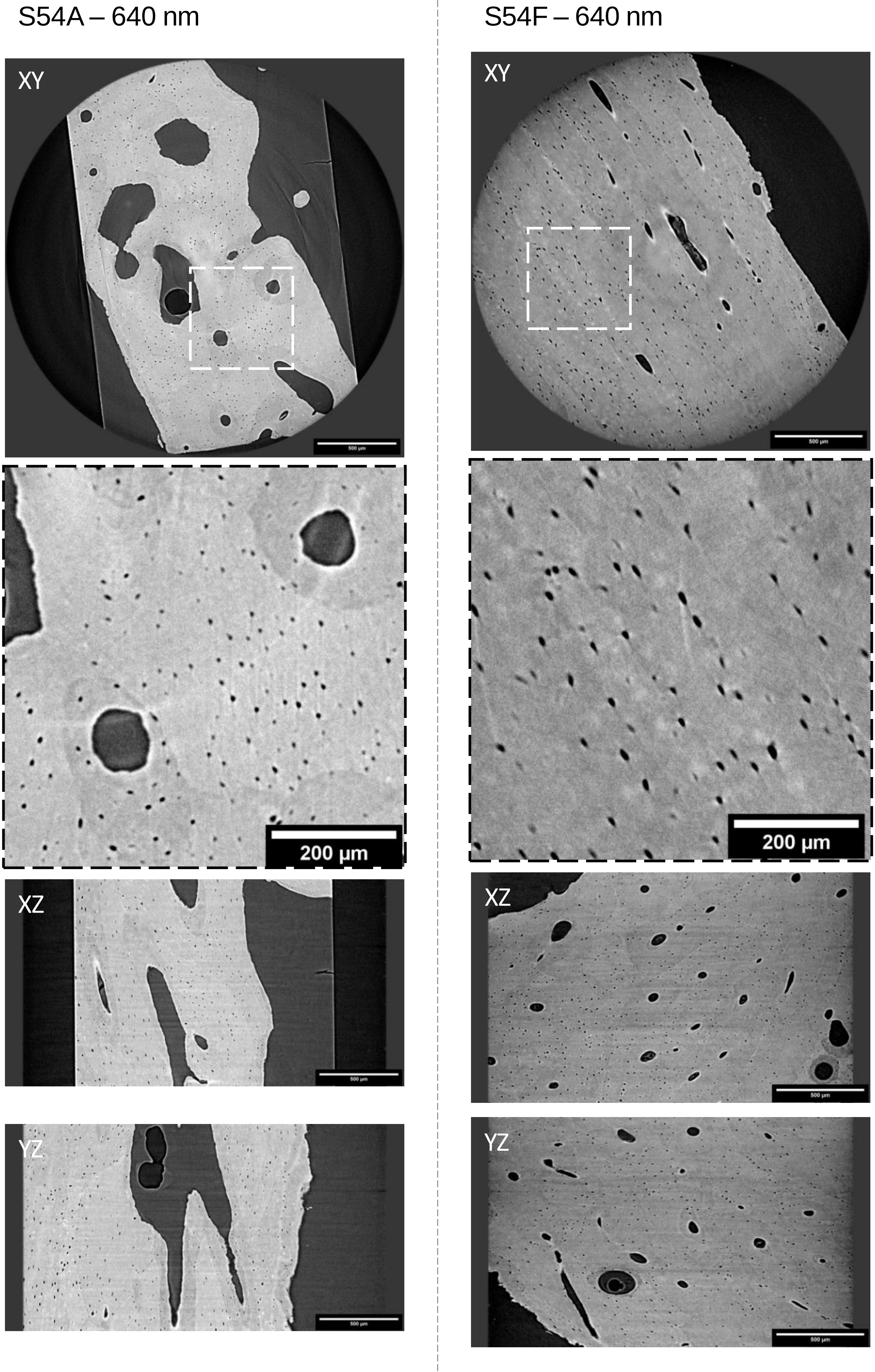
S54A – 640 nm
S54F – 640 nm
XY
XY
200 µm
200 µm
XZ
XZ
YZ
YZ

S55A – 640 nm

S55F – 640 nm

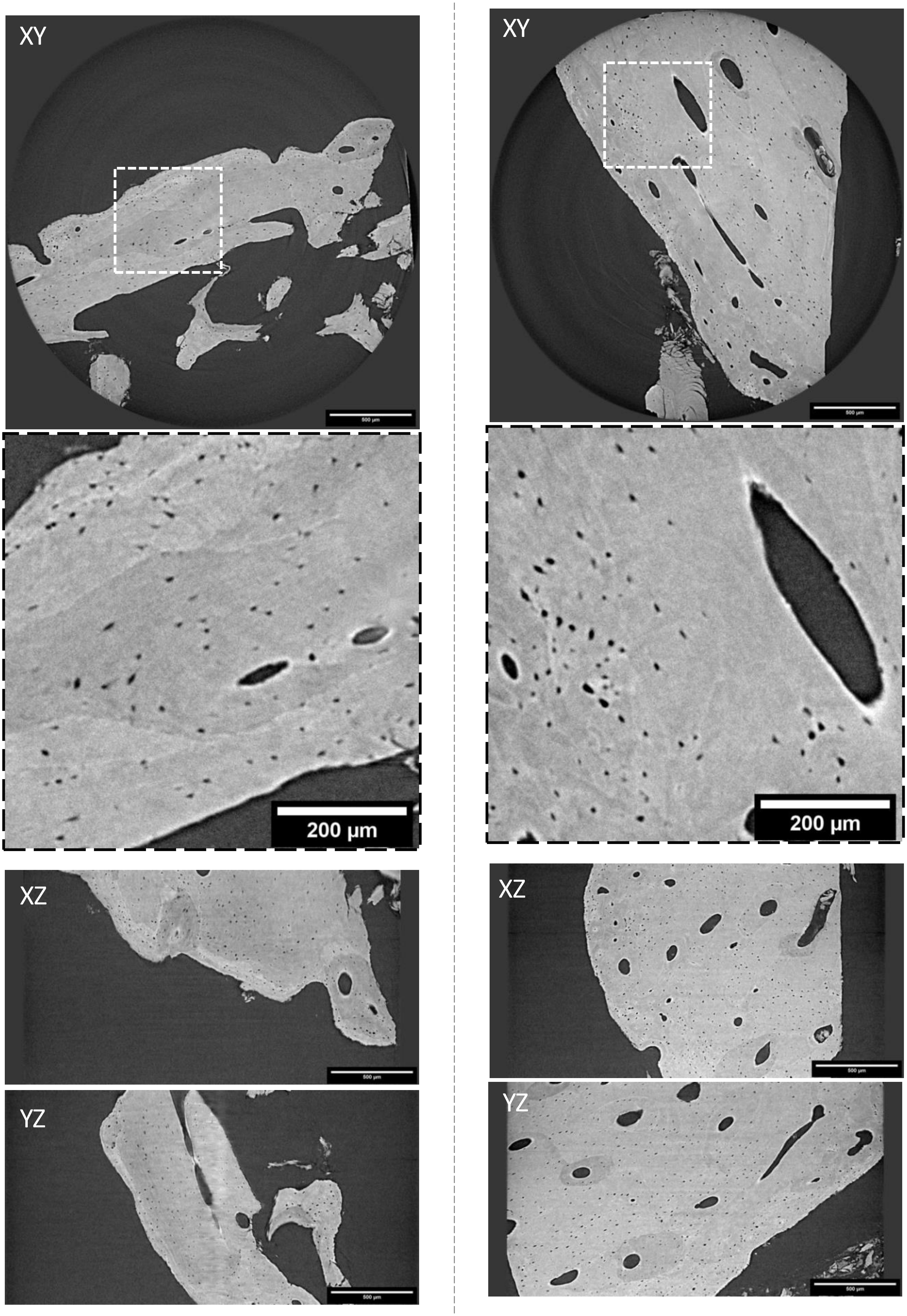

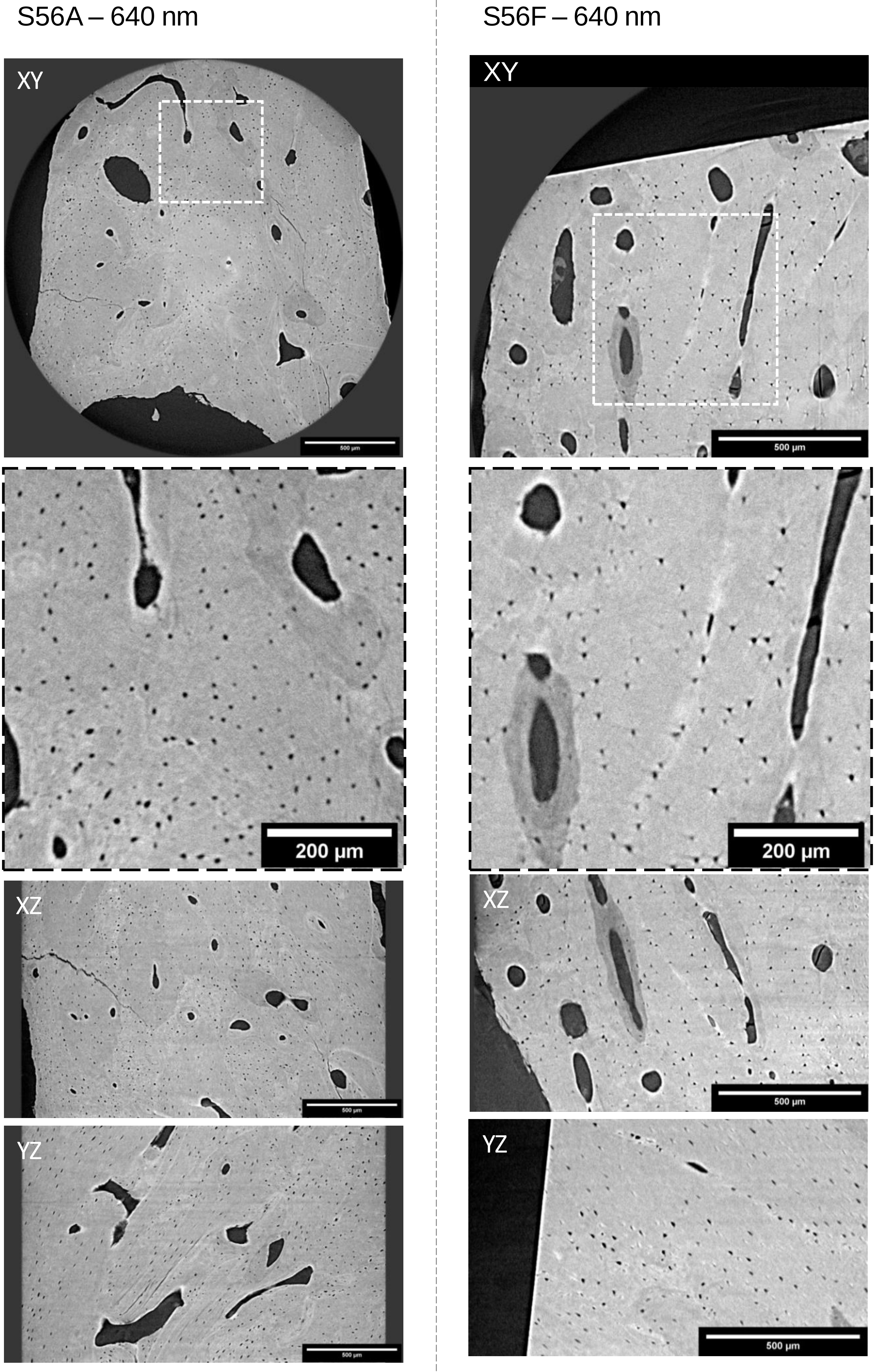
S56A – 640 nm
S56F – 640 nm
XY
500 µm
200 µm
XZ
YZ

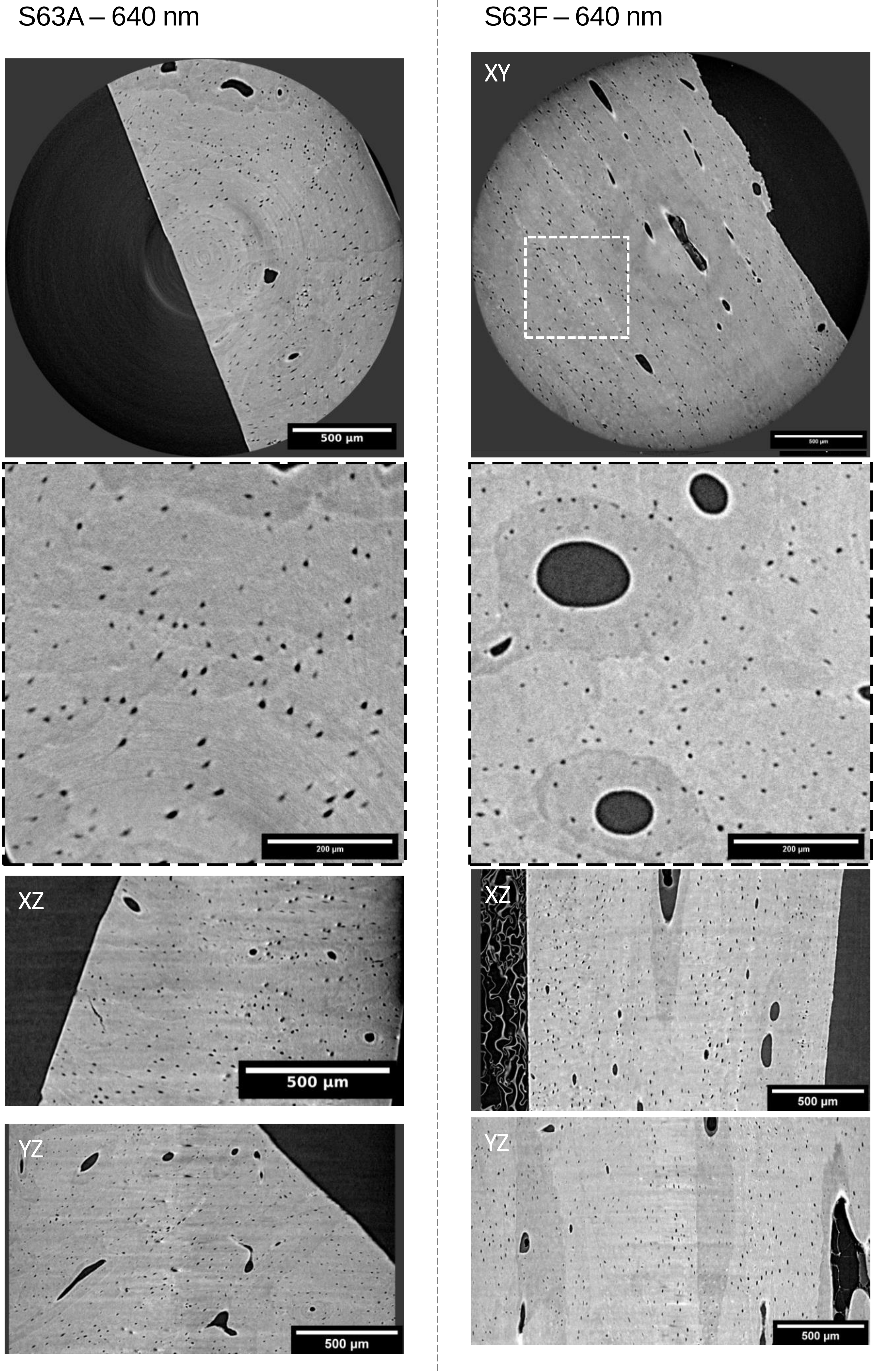
S63A – 640 nm
S63F – 640 nm
500 µm
XY
500 µm
200 µm
200 µm
XZ
500 µm
XZ
500 µm
YZ
500 µm
YZ
500 µm

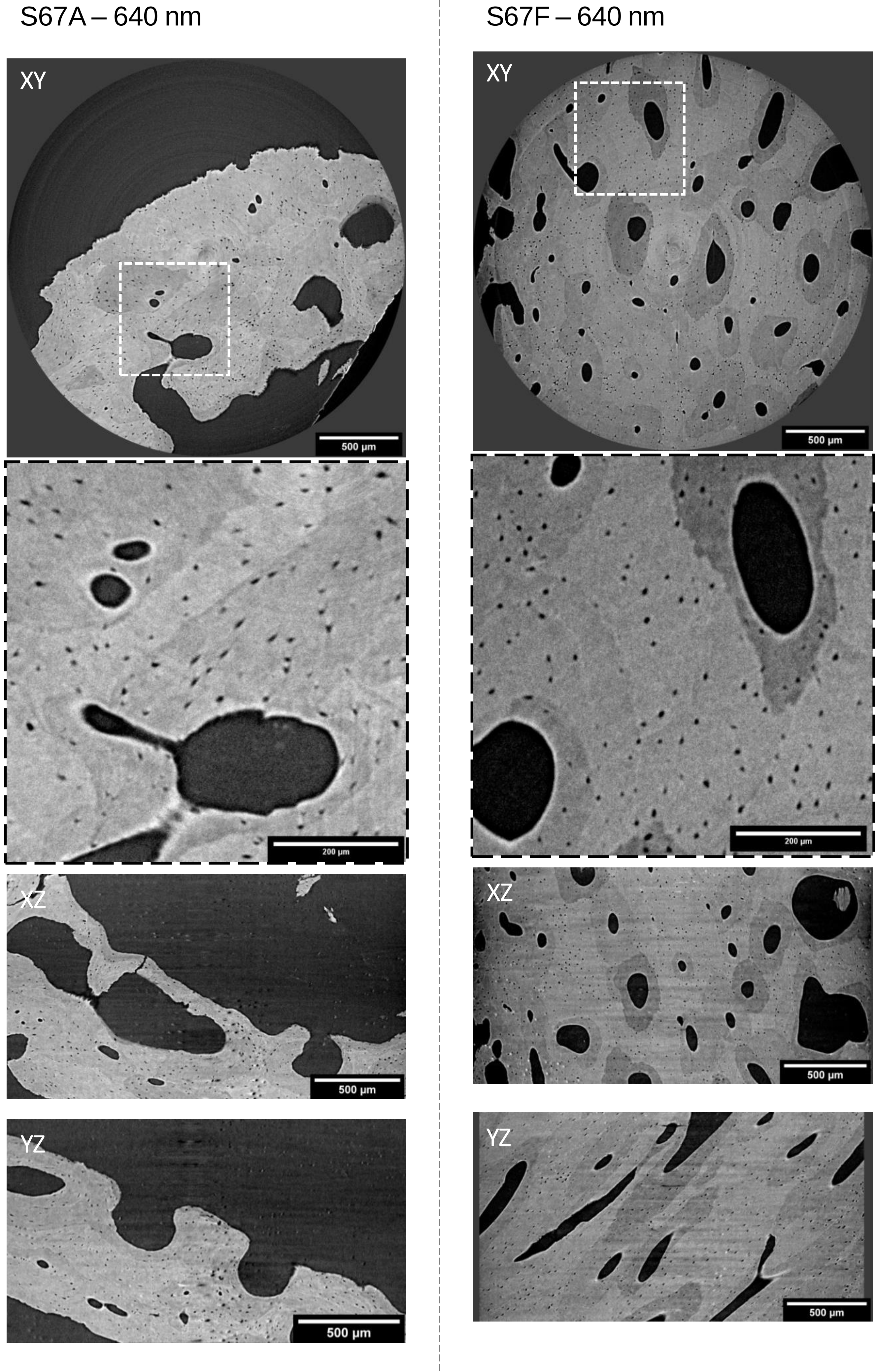
S67A – 640 nm
XY
500 µm
200 µm
XZ
500 µm
YZ
500 µm
S67F – 640 nm
XY
500 µm
200 µm
XZ
500 µm
YZ
500 µm

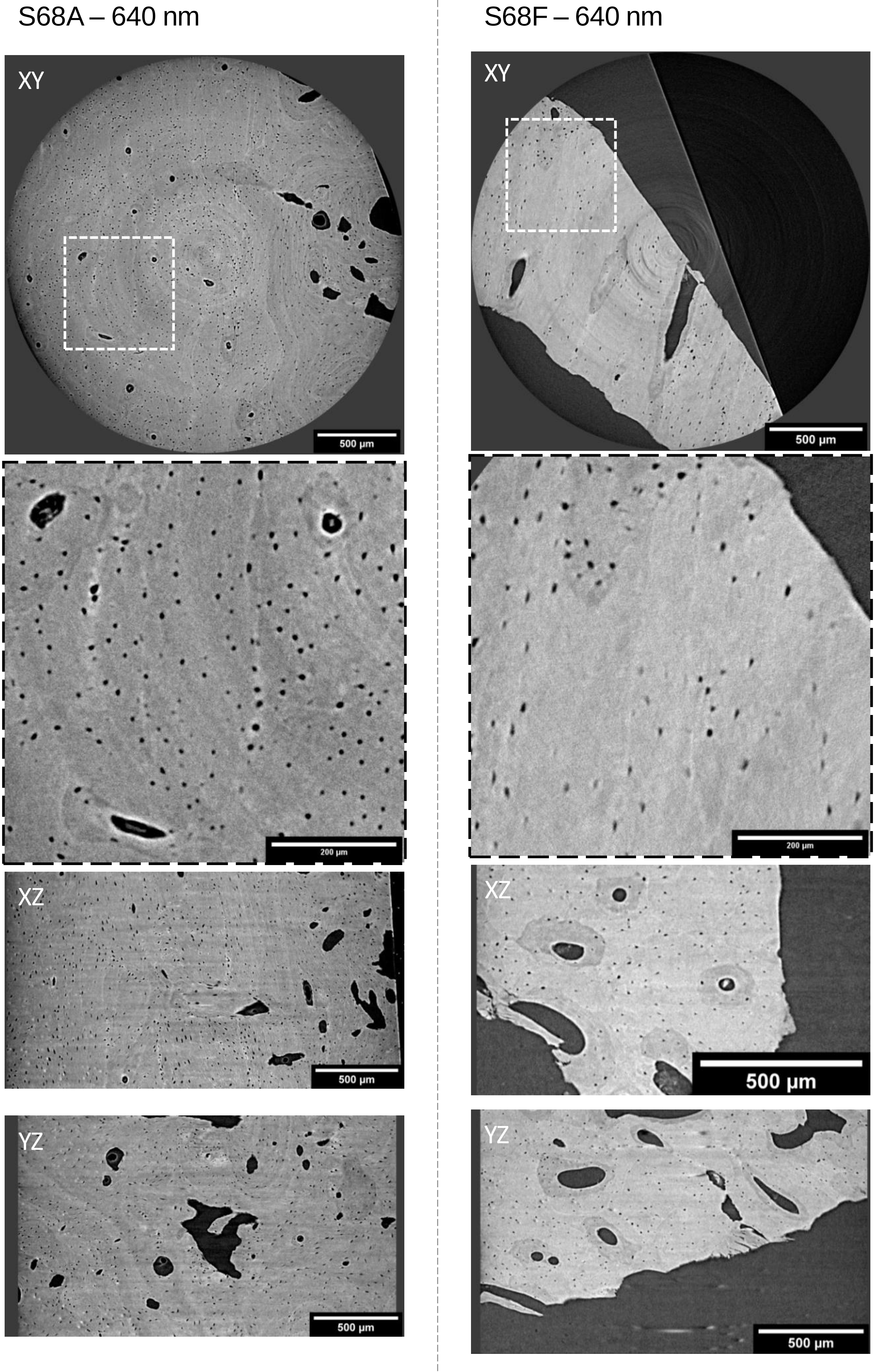
S68A – 640 nm
S68F – 640 nm
XY
500 µm
XY
500 µm
200 µm
200 µm
XZ
500 µm
XZ
500 µm
YZ
500 µm
YZ
500 µm

S69A – 640 nm

S69F – 640 nm

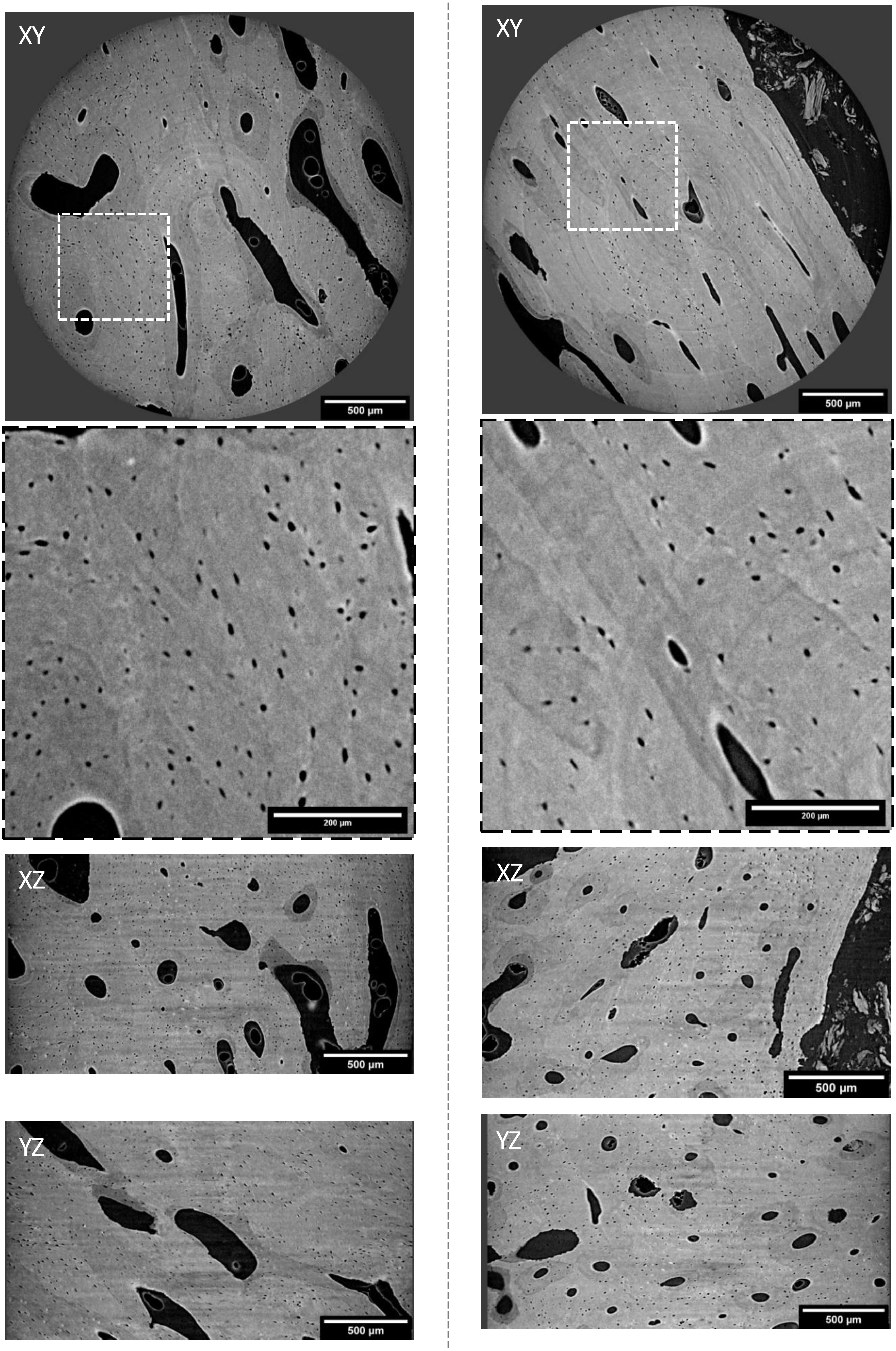

Supplementary Figure 3

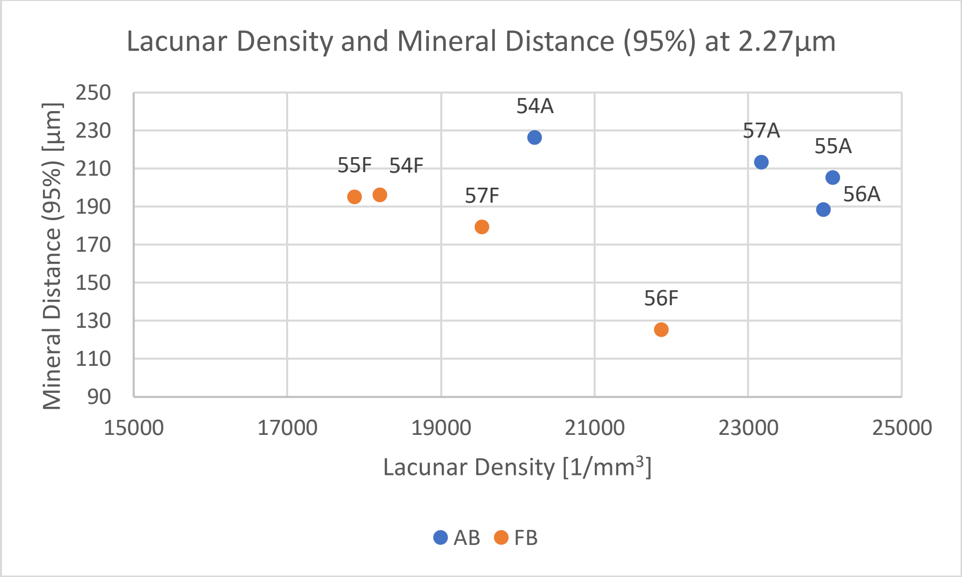

Supplement: Supplementary file 1 [file Image1.pdf]
